# What Demographic, Social, and Contextual Factors Influence the Intention to Use COVID-19 Vaccines: A Scoping Review

**DOI:** 10.3390/ijerph18179342

**Published:** 2021-09-04

**Authors:** Bara’ Abdallah AlShurman, Amber Fozia Khan, Christina Mac, Meerab Majeed, Zahid Ahmad Butt

**Affiliations:** 1 School of Public Health Sciences, University of Waterloo, Waterloo, ON N2L 3G1, Canada; baalshurman@uwaterloo.ca (B.A.A.); af6khan@uwaterloo.ca (A.F.K.); christina.mac@uwaterloo.ca (C.M.); 2 Faculty of Health Sciences, McMaster University, Hamilton, ON L8N 3Z5, Canada; majeem5@mcmaster.ca

**Keywords:** COVID-19, vaccine hesitancy, vaccine acceptance, determinants, vaccine intention, adults

## Abstract

Background: During the COVID-19 crisis, an apparent growth in vaccine hesitancy has been noticed due to different factors and reasons. Therefore, this scoping review was performed to determine the prevalence of intention to use COVID-19 vaccines among adults aged 18–60, and to identify the demographic, social, and contextual factors that influence the intention to use COVID-19 vaccines. Methods: This scoping review was conducted by using the methodological framework for scoping review outlined by Arksey and O’Malley. A search strategy was carried out on four electronic databases: PubMed, Scopus, CINAHL, and PsycINFO. All peer-reviewed articles published between November 2019 and December 2020 were reviewed. Data were extracted to identify the prevalence of, and factors that influence, the intention to use COVID-19 vaccines. Results: A total of 48 relevant articles were identified for inclusion in the review. Outcomes presented fell into seven themes: demographics, social factors, vaccination beliefs and attitudes, vaccine-related perceptions, health-related perceptions, perceived barriers, and vaccine recommendations. Age, gender, education level, race/ethnicity, vaccine safety and effectiveness, influenza vaccination history, and self-protection from COVID-19 were the most prominent factors associated with intention to use COVID-19 vaccines. Furthermore, the majority of studies (*n* = 34/48) reported a relatively high prevalence of intention to get vaccinated against COVID-19, with a range from 60% to 93%. Conclusion: This scoping review enables the creation of demographic, social, and contextual constructs associated with intention to vaccinate among the adult population. These factors are likely to play a major role in any targeted vaccination programs, particularly COVID-19 vaccination. Thus, our review suggests focusing on the development of strategies to promote the intention to get vaccinated against COVID-19 and to overcome vaccine hesitancy and refusal. These strategies could include transparent communication, social media engagement, and the initiation of education programs.

## 1. Introduction

Vaccination has been acknowledged as a significant public health achievement in decreasing the prevalence of infectious diseases since the first vaccine was invented in 1796, for use against smallpox [1]. Despite the various benefits of immunization and vaccines, the spread of misinformation and anti-vaccination movements have led to rising levels of vaccine hesitancy worldwide. In the mid-19th century, Britain had passed laws that made vaccination mandatory for children, resulting in the creation of the Anti-Vaccination League by the anti-vaccination movement in London. Since then, anti-vaccination movements have continued, resulting in a further decline in vaccination rates as the world began to witness disease outbreaks once again. For instance, the belief that the measles, mumps, and rubella (MMR) vaccine caused autism in children led to a decline in MMR vaccine uptake, even after various studies disproved the causation [2,3,4,5,6].

In November 2011, members of the Strategic Advisory Group of Experts (SAGE) recognized the reluctance to accept immunization, which affects the uptake of vaccines in both developed and developing countries [7]. Vaccine hesitancy, which can be defined as the “delay in acceptance or refusal of vaccination despite the availability of vaccine services” according to the SAGE working group, has increased worldwide due to different factors and barriers that should be addressed to improve the acceptance of global vaccination programs [8,9]. As a result, the SAGE working group established two conceptual models to understand the factors that influence the decision to accept the vaccines: the first was the “3Cs” model, composed of three approaches—complacency, convenience, and confidence—while the second was a comprehensive matrix that better portrayed the involvement of contextual, individual, and group influences alongside the vaccine-/vaccination-specific issues [8].

Following the outbreak of coronavirus disease 2019 (COVID-19), the WHO declared that the whole world faces a serious global health emergency of international concern in January 2020 [8,10]. On 11 December 2020, the U.S. Food and Drug Administration issued the first emergency use authorization (EUA) for a vaccine for the prevention of COVID-19 in individuals 16 years of age and older. The emergency use authorization allowed the Pfizer-BioNTech COVID-19 vaccine to be distributed in the U.S as the first approved COVID-19 vaccine [11]. Recent reports indicate that, as of 28 February 2021, the number of global deaths from COVID-19 reached more than 2.5 million, and the estimated number of confirmed infected cases was 114 million, making the outbreak one of the worst crises in history [12]. Therefore, global efforts and urgent actions were taken rapidly, and more than 150 countries engaged in developing safe and effective COVID-19 vaccines to help control the widespread pandemic [13,14]. At present, a dozen vaccines have been authorized and approved worldwide, and other vaccines are currently being tested in clinical trials on humans, while over 200 remain in various stages of clinical development [15]. However, previous studies have shown that the anti-vaccine movement is still increasing at a great rate, which could undermine and jeopardize researchers’ efforts to end the pandemic [16,17]. For that reason, in the era of the infodemic, it is essential that the safety and benefits of the vaccines are emphasized to the public to increase vaccine uptake. 

To date, numerous observational studies have been conducted to identify various reasons that may explain the rise of COVID-19 vaccine hesitancy and investigate the factors that affect the intention to use the vaccine [18,19,20,21,22,23,24,25,26,27,28]. As mentioned in the literature, the most common reasons that contributed to COVID-19 vaccine acceptance, hesitancy, and refusal are related to socio-demographic factors, perceived risks and benefits, and vaccine-related perceptions [13]. A systematic review was performed to provide an updated assessment of COVID-19 vaccination acceptance rates worldwide [29]; the findings revealed considerable variability in COVID-19 vaccine acceptance rates, but the authors did not provide a thorough understanding of the socio-demographic, psychological, contextual, and political factors implicated in regional and cultural differences in COVID-19 vaccine hesitancy. Another rapid systematic review aimed to explore people’s perceptions regarding COVID-19 vaccines over time and analyze factors pertaining to vaccine perceptions and intention during the pandemic [30]; however, in this review, a limited screening strategy and search terms were used, which may not address all the relevant studies; additionally, the inclusion of studies was restricted to include only surveys and questionnaires; moreover, the use of Google search to obtain a large number of related articles was another limitation of this study, as many authors argue that the usage of Google search in systematic reviews might be inappropriate, as it results in personalization of search results and, thus, leads to a form of selection bias [31,32]. Another recent scoping review investigated the vaccine hesitancy among Chinese scholars and identified a number of determinants of vaccine hesitancy in China; these determinants were categorized into four different approaches: vaccine safety, vaccine incident response, professional conduct of vaccination, and parental concern [33]; however, this review focused only on publications in China, and addressed the vaccine hesitancy in general, not specific to COVID-19 [33].

To overcome the aforementioned gaps in the existing research, we conducted a comprehensive scoping review. In addition, the scarcity of previous reviews allowed us to outline a framework that maps all of the key concepts underpinning the factors that affect the intention to use COVID-19 vaccines. This enhances the significance of our review, as this area has not previously been reviewed comprehensively. Furthermore, analyzing, summarizing, and disseminating research findings in a scoping review can provide a mechanism to policymakers and practitioners by which to visualize the depth and breadth of the intention to get COVID-19 vaccines. This scoping review also went further in identifying the factors that affect the intention to use COVID-19 vaccines based on constructs of the health belief model (HBM); this theoretical model has been widely used to assess the factors behind decision making and help in understanding what encourages and discourages people from adopting health-related behaviors [34,35]. The HBM has also been used in the context of vaccination—particularly influenza vaccination [34,36]. Consequently, conducting this scoping review helped us to scope a body of literature related to the factors that influence the intention to use COVID-19 vaccines; thus, it could be a helpful precursor to promote the identification of parameters for future systematic reviews. Likewise, the adoption of health belief model components in this review was useful to clarify significant predictors of COVID-19 vaccination acceptance, refusal, and hesitancy. On the other hand, it is worth mentioning that this review focused on adults aged 18–60 years as the target population, because several studies have reported a decline in adults’ willingness to get vaccinated against COVID-19 when compared to those above the age of 60 [19,20,37,38,39,40]. Therefore, our objectives were to determine the prevalence of intention to use COVID-19 vaccines among adults aged 18-60 years of age, and to identify the demographic, social, and contextual factors that influence the intention to use COVID-19 vaccines.

## 2. Materials and Methods

### 2.1. Overview

This scoping review was conducted by using the methodological framework for scoping review outlined by Arksey and O’Malley [41]. It also followed the Preferred Reporting Items for Systematic Reviews and Meta-Analyses (PRISMA) extension for scoping reviews’ guidelines [42].

### 2.2. Search Strategy and Selection Criteria

A comprehensive search strategy was carried out on four electronic databases: PubMed, Scopus, CINAHL, and PsycINFO. The search strategy included extensive keywords and MeSH terms for each database to cover all the articles related to the research question. Generally, the search was done using the following strategy: (vaccin* OR immuniz* OR immunis* OR inoculat* OR Moderna OR Pfizer OR anti-vaccin* OR immunization OR vaccines OR mass vaccination) AND (hesitan* OR accept* OR refus* OR preference* OR willingness OR intention OR trust OR mistrust OR attitude* OR avoidance OR distrust OR knowledge OR doubt OR fear OR perception* OR misconception* OR misinformation OR belief OR dilemma OR behavio* OR concern* OR delay OR confidence OR adherence OR nonadherence OR noncomplian* OR complian* OR uptake OR opinion* OR anxiety* OR decision* OR attitude to health OR health attitudes OR vaccination refusal OR health knowledge, attitudes, practice OR patient acceptance of health care OR trust). Additionally, CADTH’s database search strings were used to search for articles related to COVID-19, since these strings have been extensively used in published articles; thus, the CADTH database helps to conduct comprehensive literature searches for both scoping and systematic reviews (search strategy details in Appendix A). All peer-reviewed articles published between November 2019 and December 2020 resulting from these searches, along with relevant references cited in those articles, were reviewed. A list of inclusion and exclusion criteria was set out, and studies were selected according to these criteria (Section 2.6 in the methods).

### 2.3. Screening Procedure

All peer-reviewed articles published between November 2019 and December 2020 were reviewed and imported into Covidence, which was used for the screening process. Title/abstract screening and full-text screening were performed by the primary reviewer (B.A.A.) and the secondary reviewers (C.M. and A.F.K.). Disagreements were resolved by the primary reviewer (B.A.A.), and the entire scoping review process, including the search strategy and manuscript, was monitored by the tertiary reviewer (Z.A.B.).

### 2.4. Extracting the Data

All relevant publications were recorded in a spreadsheet in Microsoft Excel, including information as follows: first author, country, type of study, study population, the prevalence of intention to vaccinate, key findings and relevant factors (demographic, social, and contextual factors) (Table 1).

### 2.5. Presenting and Reporting the Results

All of the factors that influence the intention to use COVID-19 vaccines were extracted from the Excel sheet. Then, the results were presented and categorized into four main sections: (1) selected articles; (2) descriptive analysis of articles; (3) the prevalence of intention to use COVID-19 vaccines; and (4) identifying the factors of vaccine intention: demographic, social, and contextual.

All of the extracted factors were categorized according to a modified health belief model. First, modifying variables within the HBM were captured as demographic factors, such as gender, age, marriage status, race/ethnicity, education level, profession, household income, and employment status. Second, social factors were considered—those factors and sources that affect thoughts or attitude in social contexts in general situations, such as social density and pro-social concern, communication and media, socioeconomic status, social solidarity, trust in government, and political beliefs. Third, contextual factors were defined as those specific factors related to the intrapersonal or surrounding circumstances that influence the person’s behaviors or attitudes in a particular instance (e.g., the intention to take or refuse vaccines). In this review, many contextual factors were identified, including vaccine safety, vaccine efficacy/effectiveness, health history, influenza vaccination history, anti-vaccine attitudes or beliefs, vaccine recommendations by scientists, and many others.

The actual HBM consists of six main concepts: perceived susceptibility, perceived severity, perceived benefits, perceived barriers, cues to action, and self-efficacy. These concepts were modified in order to categorize the studied factors into more appropriate themes under the category of “contextual factors”. Perceived susceptibility and perceived severity were combined and referred to as “health-related perceptions”. The perceived benefits concept was replaced with vaccine-related perceptions, while cues to action was substituted for vaccine recommendations. In addition to this, we added vaccination beliefs and attitudes to investigate more related factors in-depth, as this category can be a surrogate for the self-efficacy category within the HBM. Lastly, demographics and social factors were considered to modify factors that already existed in the original HBM.

### 2.6. Inclusion and Exclusion Criteria

Throughout the title/abstract screening and full-text screening, the following inclusion and exclusion criteria were used to determine the final set of studies in the scoping review: Firstly, the inclusion criteria consisted of scholarly peer-reviewed articles that were related to vaccine hesitancy, acceptance, refusal, trust/distrust, perceptions, concerns, confidence, knowledge, attitudes, and beliefs about the COVID-19 vaccines. For predictors and exposure, studies that presented data on possible demographic, social, and contextual predictors that affect the uptake of COVID-19 vaccines were included. These studies included cross-sectional studies, case–control studies, randomized controlled trials, cohort studies, and/or qualitative studies. The criteria included studies from any country of origin with publication dates from November 2019 to December 2020. Lastly, studies that focused on adults aged 18 years old and above were included.

The exclusion criteria consisted of studies that were not related to the COVID-19 vaccine. Non-peer reviewed articles—such as editorials, perspectives, analyses, reports, preprints, letters, commentary/opinion pieces, reviews, conference articles, essays, and pilot studies—were excluded from the final study selection. Studies that were focused on safety and vaccine development research—such as immunogenicity and serological studies, preclinical trials, and efficacy trials—were excluded. Furthermore, cost–benefit analysis or cost-effectiveness studies, along with animal vaccination studies or trials, were also excluded. Views of the public’s or healthcare workers’ recommendations were also an exclusion criterion. Lastly, studies that were not in the English language and not available as full-text articles were excluded from the final study pool.

## 3. Results

### 3.1. Selected Articles

**Figure 1:**
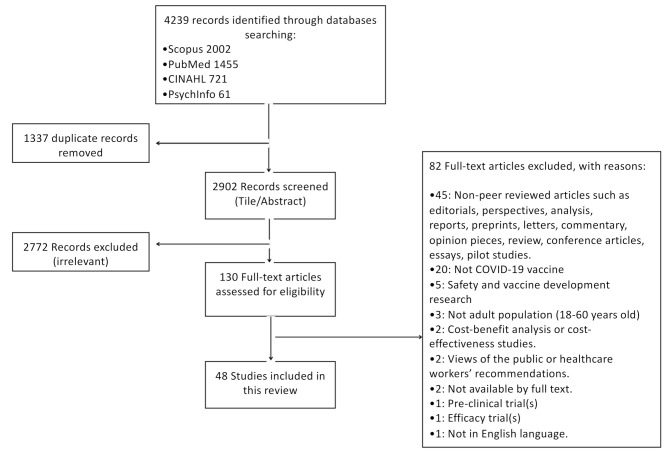
Preferred Reporting Items for Systematic Reviews and Meta Analyses (PRISMA) flow chart of article extraction from the literature search.

A total of 4239 studies were identified through the database searches (Scopus (2002 articles), PubMed (1455 articles), CINAHL (721 articles), and PsycINFO (61 articles)), from which 1337 duplicates were removed, resulting in 2902 studies included in the screening process. After title and abstract screening, 2772 were excluded because they were irrelevant to the research question. Out of the remaining 130 full-text articles, 82 papers were excluded based on the exclusion criteria (Figure 1). Finally, 48 relevant articles were identified for inclusion in the review.

### 3.2. Descriptive Analysis of Articles

The 48 included studies were conducted in various countries around the world. The majority (27%) of these studies were conducted in the USA (27%; 13/48) [19,21,23,43,44,45,48,50,53,56,65,67,75], followed by China (12.5%; 6/48) [46,54,55,57,63,69], the UK (12.5%; 6/48) [39,51,52,60,65,68], and Italy (12.5%; 6/48) [20,37,47,59,64,70]. The rest were conducted in European countries (France, Spain, Ireland, and Germany), Canada, the Democratic Republic of Congo, Indonesia, India, Turkey, Saudi Arabia, Australia, Mexico, Malta, Israel, Mexico, and Malaysia. Most of the studies were cross-sectional surveys, except for four studies, which comprised discrete choice experiments (4%; 2/48) [55,63], a follow-up experiment (2%; 1/48) [48], and a randomized control experiment (2%; 1/48) [67]. The majority of research did not focus on a specific risk group, but addressed the general public (73%; 35/48) [9,19,20,21,23,25,27,28,37,39,43,44,45,46,47,48,51,52,53,55,56,58,59,61,62,63,64,65,66,67,68,69,73,74,75]. Other studied risk groups were healthcare workers (health professionals, nurses, or others) (17%; 8/48) [22,24,26,38,40,54,57,71], university students and academics (8%; 4/48) [26,49,70,72], and parents and their guardians (2%; 1/48) [60]. The intention to vaccinate against COVID-19 was the main outcome variable in the majority of the studies (39.5%; 19/48) [20,21,23,26,39,40,43,48,50,54,55,57,58,59,61,63,64,66,75]; vaccine acceptance was assessed in 9 studies [19,25,27,28,38,45,46,53,60], while vaccine hesitancy was investigated in 10 studies [9,22,24,44,51,52,69,70,71,72]. Other outcomes were to assess the uncertainty and misconceptions about COVID-19, the attitudes towards potential COVID-19 vaccines, and to understand the barriers and facilitators to receiving a future COVID-19 vaccine [37,47,49,56,62,65,67,68,73,74]. 

### 3.3. The Prevalence of Intention to Use COVID-19 Vaccine

**Figure 2:**
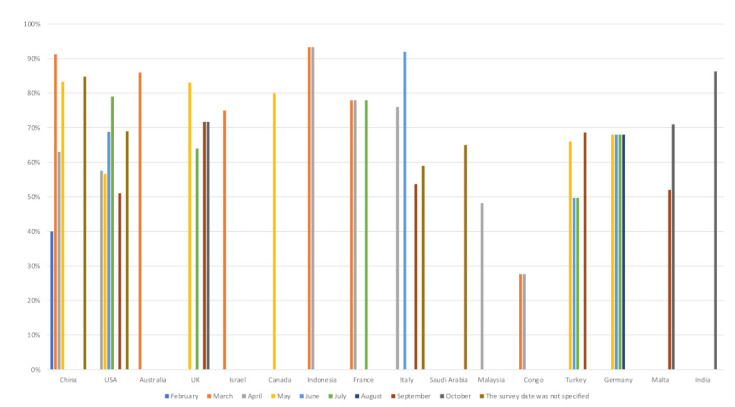
The prevalence of intention to receive COVID-19 vaccines globally.

Most of the studies (94%; 45/48) in this review reported the prevalence of intention to get a COVID-19 vaccine [9,19,20,21,22,24,25,26,27,28,37,38,39,40,43,44,45,46,47,49,50,51,52,53,54,55,56,57,58,59,60,61,62,63,64,66,67,68,69,70,71,72,73,74,75], while only three studies did not measure this prevalence [23,48,65]. The global trend of intention to get a COVID-19 vaccine showed a fluctuation in public intentions to get vaccinated over time, from February to October (Figure 2). The highest acceptance rates were noticed among adults from Indonesia (93%) [28], Italy (92%) [47], and China (91%) [46], followed by India and Australia (86%) [27,61]. On the other hand, the lowest acceptance rates were found in the Democratic Republic of Congo among healthcare workers (28%) [38], in Malta among university students and academics (31%) [72], and in China among nurses (40%) [57]. Furthermore, the majority of studies (*n* = 34/45) [9,19,20,24,25,26,27,28,39,40,43,44,45,46,47,49,50,51,52,53,54,55,56,58,60,61,62,63,66,68,69,70,71,75], reported a relatively high prevalence of intention to get vaccinated against COVID-19, with a range from 60% [75], to 93% [28].

### 3.4. Identifying the Factors of Vaccine Intention: Demographic, Social, and Contextual

#### 3.4.1. Theoretical Constructs

All factors that affect intentions to receive COVID-19 vaccines were extracted from the 48 articles. In order to identify the most relevant factors, the outcomes presented fell into 55 theoretical constructs. These were classified into seven themes: demographics, social factors, vaccination beliefs and attitudes, vaccine-related perceptions, health-related perceptions, perceived barriers, and vaccine recommendations. Themes were derived from the health belief model (HBM)—a widely used framework for guiding health promotion and disease prevention programs and understanding health-related behaviors. The actual HBM consists of six main concepts: perceived susceptibility, perceived severity, perceived benefits, perceived barriers, cues to action, and self-efficacy. These concepts were modified in order to categorize the studied factors into more appropriate themes under the category of “contextual factors”. Perceived susceptibility and perceived severity were combined and referred to as “health-related perceptions”. The perceived benefits concept was replaced with vaccine-related perceptions, while cues to action was substituted for vaccine recommendations. In addition to this, we added vaccination beliefs and attitudes to investigate more related factors in-depth, as this category can serve as a surrogate to the self-efficacy category within the HBM. Lastly, demographics and social factors were considered to modifying factors that already existed in the original HBM.

**Figure 3:**
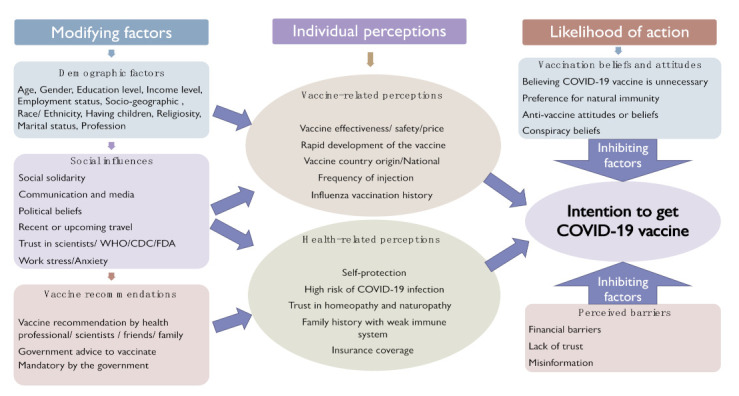
Conceptual framework for the hypothesized predictors of intention to receive COVID-19 vaccines based on the modified health belief model (HBM).

Figure 3 provides an overview of identified theoretical constructs and themes using a conceptual framework based on a modified health belief model for the hypothesized predictors of intention to receive a COVID-19 vaccine. In this review, some of the theoretical constructs and factors were significantly associated with COVID-19 vaccination beliefs and attitudes, while others were not (Table 2). Age, gender, education level, race/ethnicity, vaccine safety and effectiveness, influenza vaccination history, and protection from COVID-19 were the most prominent factors associated with intention to get vaccinated against COVID-19. Table 2 shows the studied variables that have been significantly associated with intentions to use COVID-19 vaccines in the literature; however, it does not report the actual significance values.

#### 3.4.2. Demographic Factors

All studies reported demographic factors associated with COVID-19 vaccination amongst adults older than 18 years. The most frequently assessed demographics were age, gender, education, and ethnicity. The less frequently used constructs included having children, place of work, religiosity, and smoking status. Age, gender, and education in most studies were significantly associated with intention to receive COVID-19 vaccines. Eight studies conducted at different times after the coronavirus outbreak in March, April, May, and September 2020 showed that men and older people were more likely to receive the vaccination than women and younger people [38,40,45,46,53,58,62,65,72]. A study conducted in the USA found that the risk of mortality elicits a larger proportion willing to vaccinate than morbidity alone, which explains why older populations were more willing than younger ones [76]. In contrast, five studies indicated that younger people tended to be more receptive to coronavirus vaccination, while only two studies—in China and Australia—reported that females were more likely to get the vaccine at the beginning of the pandemic [19,20,44,54,61,63,75]. One study in Italy reported that middle-aged individuals (35–59 years) had lower willingness to vaccinate for COVID-19 than the 18–34 and over 60 age groups [59]. Of note, there is a growing gap between those with low and high education levels; many studies showed that higher education level was associated with greater vaccination intention than lower education level [19,21,25,43,51,53,56], while others recorded the opposite [44,52,55].

Regarding race and ethnicity, Reiter et al., found that White people in the U.S. were five times more willing to vaccinate (67%) against COVID-19 than Black people (12%), even though Black people experienced the highest COVID-19 incidence and mortality rates in the U.S., which in turn raises serious concerns about the burden of COVID-19 [45]. Nine surveys conducted in the U.S. and UK showed that White people consistently expressed greater acceptance of COVID-19 vaccines than Black people [19,21,23,45,50,51,53,60,75]. These different levels of COVID-19 vaccine hesitancy exhibit a distinct challenge and threat to achieving health equity [75]. Other socio-demographic factors were also measured, including employment status, profession, and place of work. Medical jobs were the occupation with the highest projected vaccine uptake, followed by academics, students, and those employed in the public sector [9,24,25,28,37,38,40,58,71,73]. Additionally, low income, unemployment or retirement, living in rural settings, being married, and having children were all associated with a low percentage of intention to receive a vaccine [21,44,45,50,51,75].

#### 3.4.3. Social Factors

Ten constructs related to social factors were identified across the studies; these were: social solidarity, socioeconomic status, social density and prosocial concern, child protection/parental concerns, communication and media, trust in government, political beliefs, recent or upcoming travel, trust in scientists/WHO/CDC/FDA, and work stress/anxiety. The most common investigated factor was political leaning; three U.S.-based surveys were carried out to provide an overview of public opinion surrounding COVID-19 vaccination. All of these studies found that people with liberal political views expressed the strongest SARS-CoV-2 vaccine intentions, followed by moderates, and then conservatives [43,45,50]. This was explained by the political polarization during the pandemic, which may lead individuals with different political inclinations to have various perceptions of risk and level of threats and, thus, different notions of the necessary actions to be taken [45]. Social norms and prosocial concerns were studied by Freeman, Taylor, and Jung, who examined the interactions between social factors and vaccine hesitancy, as well as the motivational roots of this hesitancy [44,48,51]; their findings showed that people with prosocial behaviors and social contacts/activities were less hesitant to receive a vaccine compared with those with individual concern conditions. The reasoning behind this is that people with more prosocial concerns tend to protect the vulnerable members of their community and to decrease coronavirus transmission risk as much as possible [44].

Reviewing the literature, we found that three social sources were responsible for the motivation or refusal to get the vaccine: the government; the scientists, and trusted agencies such as the WHO, CDC, or FDA; and the communication and social media. A large international study showed that fake news can increase susceptibility to misinformation, which may make people less likely to report willingness to get vaccinated against COVID-19. Moreover, higher trust in scientists was associated with a 73% increase in the odds of getting vaccinated, and a 79% increase in the odds of recommending vaccination to others [65]. Another cross-sectional study in the UK concluded that reluctance to receive a vaccine was associated with the belief that the media have over-exaggerated the risks of COVID-19, and that the timeline for the outbreak will be short [68]. Malik and his colleagues reported that participants with the highest confidence in healthcare professionals (75%), the CDC (64%), and state health departments (62%) were more likely to get vaccinated against COVID-19 [53]. Likewise, Kreps et al. revealed that the FDA, CDC, and WHO recommendations to encourage people to take COVID-19 vaccine have contributed to increasing the likelihood of vaccination more than recommendations from political figures [19]. Child protection and parental concerns were also observed as predictors that influence vaccination intention. Parents were hesitant to give the vaccine to their children, as the statistical results showed that 34.3% of parents were unsure of taking the vaccine for themselves, while 41% were unsure of giving their children the vaccine [60]. Only two studies investigated work stress and its relation to vaccine uptake; the findings from both studies showed that work stress and anxiety were positively associated with intention to receive a COVID-19 vaccine [54,73].

#### 3.4.4. Contextual Factors

Five themes emerged under this category: vaccination beliefs and attitudes, vaccine-related perceptions, health-related perceptions, perceived barriers, and vaccine recommendations. These factors were reported frequently across the included studies in the scoping review.

##### Vaccination Beliefs and Attitudes

Among these constructs, the most significant ones presented in studies were vaccine confidence and vaccine hesitancy. Vaccine confidence is the trust that the person has in taking the recommended vaccine, whereas vaccine hesitancy refers to emerging concerns and hesitance to take a vaccine. According to Kowk et al., who conducted a cross-sectional survey among nurses, those who had stronger vaccine confidence were more likely to receive a COVID-19 vaccine [54]. Another study found that trust and confidence in the vaccines led to a higher probability of vaccination intention [55]. In comparison, vaccine hesitancy is associated with a decrease in intention to take COVID-19 vaccines [20,40]. A study conducted in the UK found that higher vaccine hesitancy was associated with lower adherence to all safety guidelines and a lower likelihood of getting tested [51]. Freeman et al. found that vaccine hesitancy was lower in those at very high risk of severe COVID-19 illness. In contrast, mistrust, beliefs that vaccine safety data was often fabricated, and deceit about vaccine efficacy and safety from the government and pharmaceutical companies were reasons associated with higher levels of vaccine hesitancy [51]. Similarly, anti-vaccine attitudes or beliefs were associated with higher vaccine hesitancy, resulting in lower intention to vaccinate [21]. Bertin et al. found that all types of conspiracy beliefs negatively impacted vaccine attitudes and the intention to vaccinate against COVID-19 [49]. Lastly, preference for natural immunity and believing COVID-19 vaccination is unnecessary were both associated with lower intent to vaccinate against COVID-19 [44,57]; however, these themes were poorly represented in the literature, as there is a lack of studies surrounding these topics.

##### Vaccine-Related Perceptions

Nine constructs were identified under vaccine-related perceptions; vaccine efficacy/effectiveness, vaccine safety, influenza vaccination history, and vaccine adverse side effects were the most dominant and significant in the literature. In general, studies found that higher vaccine efficacy and evidence of the vaccine’s efficacy were associated with a higher likelihood of receiving the vaccine [19,27,28,44,45,50,55,56,62,63]. When determining the factors that impact vaccination decisions, a study from the U.S. reported that 81% of participants said that the vaccine’s efficacy was a significant factor [45]. Another study in Indonesia revealed that 93.3% of adults were willing to be vaccinated with a 95%-effective vaccine, whereas 67% were willing to be vaccinated with a 50%-effective vaccine; thus, vaccine effectiveness was one of the factors to change the direction of vaccine intention [28]. In contrast, concerns about vaccine efficacy were associated with a decrease in likelihood to receive the vaccines due to worries about the accelerated vaccine development [21,27,50,51,57,60,66,69]. Trends for vaccine safety were similar to those of vaccine efficacy; overall, individuals who received evidence of the vaccine’s safety and believed that the vaccine was safe were more likely to accept the vaccine [9,27,39,44,50]. Adverse side effects from the vaccine were also an important factor in willingness to take COVID-19 vaccines. A decrease in intention to vaccinate was associated with the presence of adverse side effects and individuals who were concerned about potential adverse side effects [27,44,66,69]; this includes concerns related to insufficient information about the long-term side effects of a novel vaccine [22]. Conversely, fewer adverse side effects and the belief that vaccination would not cause side effects were associated with higher intention to vaccinate [9,19,39,55,61,63]. In addition, influenza vaccination history was a significant factor, with individuals who had previously received an influenza vaccine being more likely to receive a COVID-19 vaccine [9,19,21,39,40,46,47,53,56,57,62,63,72]. Kreps et al. stated that vaccine history was the most important predictor of COVID-19 vaccination intention [19].

For the remaining constructs, vaccine price was also a determinant of vaccine intention studied in many articles. The trends from the studies show that lower vaccine price is correlated with higher willingness to vaccinate, although it may not be the most significant factor [55,61,63,66]. Additionally, concerns surrounding the impact of the speed of development on the safety and efficacy of the vaccines were reported to be an explanatory factor for vaccine hesitancy among participants [51], although participants from an Australian study preferred the vaccine to be available in a shorter period [61]. The vaccine’s country of origin was also examined in several studies. Two studies conducted among U.S. adults revealed a strong public preference for a domestically manufactured COVID-19 vaccine (U.S.) rather than an imported one [19,56]. Surprisingly, vaccine preferences were heterogeneous in China; the Chinese population in one study showed a higher willingness to receive a foreign made rather than a national vaccine [63], while another study reported conflicting results [69].

##### Health-Related Perceptions

High risk of COVID-19 infection and fear about COVID-19 were mentioned in the greatest number of studies. Overall, those who perceived a high risk of COVID-19 infection were associated with a higher likelihood of accepting COVID-19 vaccination [9,25,28,39,40,46,51,55,64,66]. According to a cross-sectional study conducted in Saudi Arabia, those who had a higher perceived risk of infection were 2.13 times more likely to receive a vaccination compared to those with a lower perceived risk [25]. Health history/medical history, insurance coverage, and contact with COVID-19 cases were also significant factors impacting vaccination intentions. Wang et al. found that those with chronic conditions were more likely to have the intention to receive a COVID-19 vaccine [57]. A different study by Grüner et al. found that individuals who were immunocompromised or had family with compromised immune systems were more likely to intend to be vaccinated [26]. Moreover, individuals without health insurance were less likely to vaccinate, whereas those with private health insurance were more likely to vaccinate [21,45,50,56,73,75]. Finally, being sick with COVID-19, and trust in homeopathy and naturopathy, were not well supported in the examined literature. A cross-sectional study conducted in the USA found that being sick with COVID-19 was associated with an increased likelihood of intention to vaccinate [45]. In contrast, a cross-sectional study conducted in Germany reported that trust in homeopathy and naturopathy was associated with a lower likelihood of intention to vaccinate [26].

##### Perceived Barriers

Perceived barriers decrease the likelihood of vaccination and negatively impact vaccination beliefs and attitudes. In this category, there are four constructs, consisting of financial barriers, lack of trust, misinformation, and concerns about commercial profiteering. With respect to misinformation, Roozenbeek et al. administered a cross-sectional study in Ireland, the USA, Spain, Mexico, and the UK, finding that increased susceptibility to misinformation decreased compliance with health guidance, willingness to vaccinate, and likelihood to recommend the vaccine to vulnerable friends and family [65]. Furthermore, being exposed to information about COVID-19 on social media was correlated with higher susceptibility to misinformation in Ireland, the UK, and the USA [65]. Additionally, lack of trust was associated with lower intention to vaccinate [20,21,51]. According to a cross-sectional study from the USA, lack of trust was the second most common reason for responding “no” towards vaccination intention. In this study, lack of trust encompassed lack of trust towards vaccines, the government and the CDC, pharmaceutical companies, and vaccine development or testing processes, as well as reference to conspiracy theories [21]. In relation to financial barriers, 6.17% of participants in a study from the USA stated they would omit vaccination due to lack of financial resources [50]. Concerns about commercial profiteering were correlated with lower intention to vaccinate [44]. However, both financial barriers and concerns about commercial profiteering were poorly mentioned in the literature, since only one study examined each.

##### Vaccine Recommendations

Previous studies showed that a recommendation by a health professional/scientist increased the likelihood of vaccination [27,44,45,46,60]. A study in the USA showed that the percentage of adults who were more likely to get the vaccine increased from 56.6% to 61.8% if they received a recommendation from their healthcare providers [43]. Likewise, Reiter et al. found that 73% of participants responded that a doctor’s recommendation would be an important factor in their vaccination decisions [45]. Another cross-sectional study conducted in China found that those who valued their doctor’s recommendation were more likely to receive a COVID-19 vaccine as soon as possible [46]. Furthermore, Leng et al. found a positive correlation between willingness to take the vaccine and the proportion of acquaintances—including friends and family—being vaccinated [55]. Concerning the impact of government advice to vaccinate, Bokemper et al. found that endorsement by political leaders has a polarized effect, where there is an increase in vaccine confidence among supporters of that party. Among supporters of a different political party, the endorsement by political leaders tends to be ignored, or even undermine vaccine confidence [67]. However, the impact of government advice to vaccinate was only found to be significant in one out of two studies examined; therefore, this factor was poorly mentioned in the literature.

## 4. Discussion

This is the first comprehensive scoping review describing demographic, social, and contextual factors associated with COVID-19 vaccination. It enables the identification of factors related to COVID-19 vaccine acceptance, refusal, and hesitancy among adults (18–60 years). Seven interconnected themes on the basis of our modified health belief model framework were identified, including demographics, social factors, vaccination beliefs and attitudes, vaccine-related perceptions, health-related perceptions, perceived barriers, and vaccine recommendations. The adoption of the health belief model was uniquely modified in this review, as some of the original categories were replaced by others to fit appropriately with our research question. Demographic variables were used as a separate category that interacted with the HBM as well as the social influences category. These two categories were considered to be important factors that influence health behaviors, such as an individual’s perceived threat of sickness or disease, or even a new treatment. Meanwhile, the six main components of the HBM—perceived susceptibility, perceived severity, perceived benefits, perceived barriers, cues to action, and self-efficacy—were renamed and classified into five themes under a major category termed “contextual factors”. Perceived susceptibility and perceived severity (which are the two dimensions of “threat”) were combined and referred to as “health-related perceptions”. The perceived benefits concept was replaced with vaccine-related perceptions, while cues to action was substituted for vaccine recommendations. In addition to this, the vaccination beliefs and attitudes category was added to investigate more related factors in-depth, as this category can act as a surrogate for self-efficacy within the primary HBM.

In our review, both demographic and contextual factors were mentioned in nearly 96% (46/48) of the reviewed articles, whereas social factors were only reported in 75% (36/48) of the chosen articles. In line with findings from previous reviews, our scoping review showed a wide variability in the rates of COVID-19 vaccine acceptance, refusal, and hesitancy across different countries and subgroups, including healthcare providers and parents [29,30]. However, a relatively high tendency toward acceptance was observed in China, European countries, and North American countries. In contrast, the lowest acceptance rates were reported in the Democratic Republic of Congo, followed by Malaysia. Moreover, a recent study reported high refusal and hesitancy rates among the Middle Eastern population [77]. This could be explained by the lack of transparent health communication and effective tools in the developing countries, which are needed to reformulate the decision-making process and to provide a better understanding of the benefits and risks of vaccination, thus facilitating optimal vaccine uptake [78]. Other explanations could be related to several misconceptions that work against vaccination in developing countries. For instance, there are such socio-cultural issues in some societies, as they believe that the vaccines are given to developing countries because they have excessive production of children, and that this is a way to make them infertile (the infertility myth). Another misconception is that pharmaceutical companies encourage politicians to convince people to take vaccines in order to achieve their own financial benefits. Notably, these misconceptions arise from the notion of politicization—especially during the COVID-19 pandemic—and the poor communication between scientists and the general population.

In this review, the underlying factors that influence the intention to receive COVID-19 vaccines varied significantly by national- and the individual-level preferences, and were attributed to complex socio-demographic, psychological, and contextual influences. Demographic variables such as age and gender were the most reported factors, but also the most inconsistent predictors of COVID-19 vaccination. Although most of the included studies showed that men and older individuals were more willing to get vaccinated [38,40,53,58,62,65,72], a few studies reported the opposite results, as women and younger adults showed stronger intention to receive a vaccine [54,63,73,75]. Men are usually seen as being more receptive to vaccines than women because of some barriers and social norms that affect women and their decisions. Justifications for such a situation were reported by the WHO SAGE group, as women—most of the time—considered themselves responsible for the consequences of their decisions on their families which, in turn, decreases their confidence in the use of immunization services [79]. In addition, the control of women’s mobility by other family members, their education level, gender inequality, women’s disempowerment, and poverty alleviation are all correlated with women’s concerns about accessing healthcare and, therefore, associated with their decision making in the context of vaccination status [79]. Generally, older adults are more concerned about being infected with COVID-19 because they believe that they are more likely to require hospitalization if they contract COVID-19. In addition, the risk of COVID-19 infection and death increases with age group, which affects older adults’ decisions about getting vaccinated. In contrast, low risk perception and the perception of safety around healthy young adults discourage them from getting vaccinated [59]. Healthcare workers were also found to have higher acceptance rates than other professions because they are the first line of defense in combating the virus and, thus, most susceptible to being infected [22,26,38,58,73]. Interestingly, other demographic factors such as education level, race, and income levels were consistent across many studies. People with low educational levels, Black ethnicity, and low income levels were generally less likely to take the vaccines [19,21,43,45,47,50,51,61]. Health disparities that relate to these demographic and racial issues are a factor in the lower vaccine acceptance rate. Thus, overcoming these racial inequalities and disparities will be key to distributing COVID-19 vaccines among various communities. These disadvantaged and minority groups should be prioritized in the vaccine distribution process to improve equity.

With respect to the sources from which people get their knowledge about COVID-19, we noted a dichotomy of two major sources through the studies: The first group of people rely on trusted information from either the government, the CDC, or the WHO [19,53,55]. In comparison, the second group use social media as their primary source of information [26,62,68]. Our findings allude to the significance of source selection, as it can be associated with the likelihood to accept or refuse the vaccine. The problem of the misinformation and fake news from social media is considered to be one the biggest barriers that stand against building trust between scientific research and the general population [65]. Uniquely, our review emphasizes the effect of socio-political factors on attitudes towards the vaccines. Based on previous studies that indicated that people’s ideologies and worldviews strongly influence their perceptions and acceptance of risk, our findings investigated the effects of political leaning on the intention to receive COVID-19 vaccines [19,53,55,80,81]. In line with the findings of a nationally representative survey in the U.S. about vaccinations for flu, pertussis, and measles, our study showed that political conservatives were less likely to get vaccinated than liberals [80]. The reason for this could be that conservatives’ leaders have publicly and repeatedly expressed anti-vaccination opinions in their attempts to link childhood vaccines to autism [82]. Many studies in this review point to the major role of the government in making COVID-19 vaccination one of the leading public health interventions [26,53,62,67]. Moreover, among the main social influencers to get the vaccine are family, friends, and healthcare providers. Most of the studies emphasized the role of family and peers in convincing individuals to accept COVID-19 vaccines [27,46,60,66,69]. As a result, healthcare providers can help with the implementation of COVID-19 vaccine programs to increase the vaccination coverage rates for their patients.

A number of contextual variables have been associated with people’s behaviors with regards to COVID-19 vaccination. While there is an overlap between the constructs related to vaccination beliefs and attitudes, health-related perceptions, and other constructs, these factors are tightly bound to vaccine acceptance, refusal, and hesitancy. Vaccine efficacy/effectiveness, vaccine safety, and adverse vaccine side effects are the most influential factors that affect people’s willingness to vaccinate [9,21,27,39,46,47,50,51,57]. Consistent findings were observed in a systematic review concerning the flu vaccine [83]. There was a general consensus among all of the included studies that influenza vaccination history was a strong motivator to accept a COVID-19 vaccine [9,21,39,46,47,57]. On the other hand, the speed of developing COVID-19 vaccines was an impediment to their acceptance [9,44,51,60,61]. Although clinical trials have maintained rigorous testing, many individuals are still concerned about the speed of vaccine development and its impact on the vaccines’ efficacy and safety [9,44,51,60,61]. Of note, the distrust in healthcare systems was another crucial factor that determined the choice of many to be vaccinated or not [20,21,51]. Many previous studies have assigned the responsibility for vaccine hesitancy to conspiracy beliefs, which were substantially and negatively related to the intention to be vaccinated against COVID-19 [20,21,49,51,65]. These beliefs revolve around the politicization of COVID-19, the fabrication of vaccine safety data, the manufacturing of antibody testing to harvest people’s DNA, and the harmful effects of vaccines on children, resulting in the erosion of people’s trust [20,21,49,51,65]. Child protection was found to be the primary reason that parents were more hesitant to vaccinate their children than themselves [60]. Hence, our findings indicate the necessity of increasing parents’ knowledge about the vaccines by ensuring effective communication between the parents and their healthcare providers.

Notably, a number of studies documented that people feel the need to take the vaccines to protect themselves, their families, and their communities from catching the virus [26,60,62,64,68]. Thus, it is considered critical to enhance the notion of the prosocial benefits of vaccination and herd immunity among people to strengthen their intentions to vaccinate [48]. Accordingly, this can help to reduce the transmission of the disease not only at the individual level, but also in the community as whole. Only a handful of studies have attempted to establish a correlation between testing positive for COVID-19 and the intention to be vaccinated against it, and the results have been ambiguous. Callaghan et al. found that individuals who have been tested for COVID-19 are 68% less likely to refuse vaccination [50], while similar findings were reported by Reiter et al. [45]. Conversely, results from another two surveys reported that being infected with COVID-19 was not significantly associated with willingness to take the vaccines [39,69]. One possible explanation is that people who have been infected with COVID-19 assume that they now have a natural immunity against COVID-19, with a lower chance of getting infected again, thus making them less inclined to take the vaccines. Lastly, it is worth mentioning that the origin of vaccine manufacturing seems to affect people’s inclinations to take or refuse the vaccine. Strong preference was observed for receiving a U.S.-made vaccine rather than a Chinese one [19,56,63]. Interestingly, a previous study indicated public skepticism with regards to the effectiveness of nationally manufactured health products in China [84].

### Strengths and Limitations

Our review enabled us to conceptualize the influence of demographic, social, political, and contextual variables on acceptance of COVID-19 vaccines by using a comprehensive framework built on the constructs of the health belief model. This enhances our findings to generate more in-depth explanations and better describe the landscape of the studied phenomena compared to previous reviews. Moreover, we used an exhaustive search strategy including possible keywords and MeSH terms that related to our research question, which allowed us to cover a large number of the relevant studies tackling the subject of this review. It is also important to note that even though our topic focused on the intention to use COVID-19 vaccines, high terminological diversity—including vaccine hesitancy, acceptance, willingness, and refusal—was utilized in the screening process. However, the results of this scoping review should be interpreted with caution, since it still has some limitations. First, most of the included studies were conducted before the authorization of any COVID-19 vaccine; therefore, people’s opinions may have changed over time, particularly during this unstable pandemic. Second, we excluded non-peer-reviewed literature such as public opinions and grey literature, which previous studies have suggested should be used within reviews to foster a balanced picture of available evidence. Third, using a modified health belief model with modified components rather than the original one could result—unintentionally—in ignoring some of the important factors that relate to our research question. Fourth, no quality assessment was conducted, as would be the case in systematic reviews. Finally, literature that was not written in English was excluded, which could result in reducing the quality and the generalizability of the results. 

## 5. Conclusions

Vaccine hesitancy is a serious challenge in the fight against COVID-19, because attaining herd immunity is dependent on the vaccines’ effectiveness and the population’s intention to accept them. Our review offered an overview of various factors, themes, and constructs associated with intention to vaccinate against COVID-19 among adult populations. Consistent with the literature, demographic, social, and contextual factors are likely to play major roles in any targeted vaccination programs, especially COVID-19 vaccination. Multiple factors—including age, gender, education level, race/ethnicity, vaccine safety and effectiveness, perceived disease risk, influenza vaccination history, and vaccine recommendations by health professionals—could influence people’s intentions and, ultimately, their decisions to accept COVID-19 vaccines or not. It is possible that it is not only misinformation that affects people’s decisions to reject vaccines, but also the lack of tools with which to restructure the decision-making process and provide a clearer understanding of the vaccines’ benefits and risks. Therefore, our research suggests focusing on developing strategies to promote the intention to get vaccinated against COVID-19 and to overcome vaccine hesitancy and refusal. One potential strategy could be to increase the transparency of communication between the researchers and the general population, considering the wide variations in public beliefs about the vaccine efficacy and safety. Furthermore, the media—and especially social media—will play a key role in shaping and manipulating public attitudes to COVID-19 vaccination. Thus, it could be very effective to engage social media to establish vaccine confidence and share positive examples of vaccine acceptance. Finally, educational programs can be also considered as an effective intervention to portray the potentially serious illness that could develop from COVID-19. Subsequently, it is recommended that health authorities supplement these programs using evidence-based cues to increase people’s awareness about COVID-19 vaccines.

## Figures and Tables

**Table 1:** Data extraction table for the review.

							Key Findings and Relevant Factors	Key Findings and Relevant Factors	Key Findings and Relevant Factors
Study #	First Author	Country	Type of Study	Study Population	Aim of the Study	The Prevalence of Intention to Vaccinate	Demographic Factors	Social Factors	Contextual Factors
1	Head, K.J. (2020) [43]	USA	Cross-sectional study	Adults over 18	To determine the SARS-CoV-2 vaccine behavioral intentions of adults in the U.S, and what factors are associated with SARS-CoV-2 behavioral intentions.	56.6% likelihood without provider recommendation (41.9% very likely, 14.7% somewhat likely), 61.8% likelihood with provider recommendation (49.2% very likely, 12.6% somewhat likely)	Age, religion, employment, education, race, gender, relationship status, parenthood, and income level were measured. Less likely to vaccinate: Less education, those work in healthcare	Politics measured. Liberals more likely than conservatives	Fear and hesitancy of vaccine were measured. More likely to vaccinate: Those with high perceived threat to physical health, and those who perceived COVID-19 to be a major problem in the community
2	Taylor, S. (2020) [44]	USA, Canada	Cross-sectional study	Adults over 18	To identify the prevalence of vaccination hesitancy for a SARS-CoV2 vaccine, the motivational roots of this hesitancy, and the most promising incentives for improving the likelihood of vaccination uptake	American respondents (75%), Canadian respondents (80%)	Less likely to vaccinate: Female gender, age, completed full or partial college education, unemployed, minority status; More likely to vaccinate: Religious leaders in community recommended vaccination	More likely to vaccinate: (1) Helped protect vulnerable (2) Members of community (3) Required to attend social or sporting events (4) Received some other incentive (e.g., discount coupon) (5) Promotion by trusted news source, President/Prime Minister, or social media(6) Trust in health authorities	Less likely to vaccinate: (1) Mistrust of vaccine benefit (2) Worry about negative effects (3) Concerns about commercial profiteering (4) Preference for natural immunity; More likely to vaccinate: (1) Evidence that vaccine is safe and efficacious (2) Requirement for job (3) Required by government (4) Recommended by healthcare worker (5) Not being exploited by the pharmaceutical industry (6) Know someone with COVID-19 (8) Know someone hospitalized because of COVID-19
3	Reiter, P.L. (2020)[45]	USA	Cross-sectional study	Adults over 18	To examine the acceptability of a COVID-19 vaccine among a national sample of adults in the US	69% willing to get the vaccine (48% definitely willing, 21% probably willing)	Factors measured: Age, gender, race/ethnicity, marital status, education level, household income, religiosity, sexual identity, urbanicity, and region of residence. More likely to vaccinate if: (1) Income of USD 50,000–89,999 or USD 90,000 or more (2) Age; Less likely to vaccinate: (1) Female (2) Non-Latinx Black; (3) Lower incomes	More likely to vaccinate: Moderate or liberal in political leaning; Less likely to vaccinate: Conservative in political leaning	More likely to vaccinate: (1) Private health insurance (2) Personal COVID-19 infection (3) Healthcare provider recommendation (4) Perceived likelihood of getting COVID-19 in the future (5) Perceived severity of COVID-19 infection (6) Effectiveness of COVID-19 vaccine (7) Personal health history (8) Number of people getting infected with COVID-19 (9) Recent/upcoming travel outside of US (10) Duration of protection; Less likely to vaccinate: (1) Perceived potential harms of COVID-19 vaccine (2) No health insurance (3) Potential side effects
4	Kabamba Nzaji, M. (2020) [38]	Democratic Republic of the Congo	Cross-sectional study	Healthcare workers over 18	To estimate the acceptability of a future vaccine against COVID-19 and associated factors if offered to Congolese healthcare workers	27.7% of healthcare workers	More likely to vaccinate: (1) Male (2) Older age (3) Being a doctor; Factors measured: Age, gender, marital status, year of experience, residence, category of residence	No social factors recorded	More likely to vaccinate: (1) Positive attitude towards COVID-19 (2) Believe that isolation and treatment of people infected are effective to reduce spread of virus; Factors measured: Heard about COVID-19, attended lectures/discussions about COVID-19, knowledge towards COVID-19, attitudes toward COVID-19, practice toward COVID-19
5	Harapan, H. (2020) [28]	Indonesia	Cross-sectional study	Adults	To assess the acceptance of a 50% or 95% effective COVID-19 vaccine among the general population in Indonesia	93.3% willing to be vaccinated for a 95% effective vaccine; 67% willing to be vaccinated for a 50% effective vaccine	Factors measured: Age, gender, education level, religion, marital status, monthly income, profession, employment status, and type of urbanicity; More likely to vaccinate for 95% effective vaccine: Healthcare worker; Less likely to vaccinate for 95% effective vaccine: Retired	No social factors recorded	More likely to vaccinate: (1) Higher perceived risk (more likely to accept the vaccine, but only for the 95% effective vaccine) (2) Higher vaccine efficacy
6	Wang, J. (2020) [46]	China	Cross-sectional study	Adults over 18 in mainland China	To evaluate the acceptance of COVID-19 vaccination in China and give suggestions for vaccination strategies and immunization programs	91.30%	More likely to vaccinate ASAP: (1) Male (2) Married; Factors measured: Age, gender, marital status, education level, employment status, family income, location, region (urban vs rural)	No social factors recorded	More likely to vaccinate: (1) Vaccinated against influenza in the past season (2) If vaccine was successfully developed and approved (3) Doctor’s recommendation (4) Vaccine convenience (5) Vaccine price (6) Perceived high risk of infection (7) Believe vaccination is effective for prevention and control (8) Confirmed cases in area; No differences observed in domestic/imported vaccines, immunization schedules, wanting to receive vaccine ASAP or wait
7	Biasio, L.R. (2020) [47]	Italy	Cross-sectional study	Adults over 18 interested in looking for information about future COVID-19 vaccines	To assess people’s abilities to collect and understand information about vaccinations during the early stage of COVID-19 vaccine development	92%	Factors measured: Age, gender, residence area, employment status, and education level. Positive beliefs about vaccination were correlated with older age and higher education	No social factors recorded	Receiving seasonal influenza vaccine and perceptions regarding future COVID-19 vaccines were indicators for high level of vaccine literacy; Factors measured: Vaccine safety/efficacy, vaccine payment, children vaccination, vaccination intent for other diseases
8	Fisher, K.A. (2020) [21]	USA	Cross-sectional study	Adults in the US	To assess intent to be vaccinated against COVID-19 and identify predictors of and reasons for vaccine hesitancy	57.60%	Less likely to vaccinate: (1) Younger age (< 60) (2) Black race (3) Lower educational attainment (4) Rural setting; Factors measured: Age, gender, employment status, annual household income, marital status, household size, geographic location, setting (urban vs. rural)	No social factors recorded	Less likely to vaccinate: (1) Not having received an influenza vaccine (2) Need additional information (3) Anti-vaccination attitudes (4) Not trusting entities involved in vaccine development, testing or dissemination (5) Concerns about safety or effectiveness
9	La Vecchia, C. (2020) [37]	Italy	Nationally representative survey	Ages 15–85	To describe the attitudes towards influenza vaccination and a potential COVID-19 vaccine in Italy	53.7% (20.4% certainly, 33.3% probably)	Factors measured: Age, gender, profession, and geographic area. More likely to vaccinate: Older age (aged 55 or older), professionals, managers, and teachers (vs. office workers, merchants, farmers, and manual workers)	Factors measured: Socioeconomic status	Factors measured: Influenza vaccination history. No significant contextual factors recorded
10	Jung, H. (2020) [48]	USA	Study 3: follow-up experiment with an online survey	Adults	To examine whether prosocial concern interacted with social density, having an effect on the intention to vaccinate against COVID-19	The percentage was not measured	No demographic factors were recorded	More likely to vaccinate against COVID-19: Participants in the prosocial concern condition with low-density condition; Less likely to vaccinate: Participants in the individual concern condition with low-density condition; No difference: In the high-density condition, intentions were similar across the prosocial and individual concern conditions	No contextual factors were recorded
11	Bertin, P. (2020) [49]	France	Cross-sectional study	undergraduate students	To examine the relationship between COVID-19 conspiracy beliefs, vaccine attitudes, and the intention to be vaccinated against COVID-19	approximately 78%	Age and gender were not significantly associated with intention to get COVID-19 vaccine	Political orientation was not significantly associated with intention to get COVID-19 vaccine	conspiracy beliefs were negative predictors of intention to get vaccinated against COVID-19
12	Callaghan, T. (2020) [50]	USA	Cross-sectional study	n/a	To provide an overview of the public opinion surrounding COVID-19 vaccination that includes potential correlates and justification for intended vaccine refusal	68.87%	Factors measured: Age, gender, race, education, and income level. Less likely to vaccinate: (1) Black race (2) Women (3) High religiosity	More likely to vaccinate: Wealthier; Less likely to vaccinate: (1) Conservatives (2) Intend to vote for President Trump in 2020 (3) Lack of financial resources (4) Trust experts	More likely to vaccinate: (1) Have been tested for COVID-19 (2) View vaccines as safe, effective, and/or important; Less likely to vaccinate: (1) Do not think the vaccine will be safe or effective (2) Lack of insurance (3) Believe they already contracted COVID-19
13	Sherman, S.M. (2020) [39]	UK	Cross-sectional study	Adults over 18	To investigate factors associated with intention to be vaccinated against COVID-19	64%	Factors measured: Age, gender, ethnicity, religion, employment status, highest educational/professional qualification, total household income, region, and household number. More likely to vaccinate: Older age	More likely to vaccinate: Lower endorsement of notion that only people who are at risk of serious illness should be vaccinated, trust in the government; Less likely to vaccinate: Believe that only those at risk should be vaccinated	More likely to vaccinate: (1) Vaccinated against influenza last winter (2) Perceive greater risk for COVID-19 (3) More positive COVID-19 vaccination beliefs and attitudes (4) Weaker beliefs that vaccination would cause side effects or be unsafe (5) Informed decision; Factors measured: Living with someone vulnerable to COVID-19, personal history of COVID-19, knew anyone with COVID-19, attitudes and beliefs towards COVID-19
14	Sharun, K. (2020) [27]	India	Cross-sectional study	Adults over 18	To analyze the beliefs and barriers associated with COVID-19 vaccination among the general population in India	86.30%	Factors measured: Age, gender, education, and region. No significant demographic factors recorded	No significant social factors recorded	More likely to vaccinate: (1) Safety and effectiveness confirmed using further studies (2) Recommended by physician (3) Mandatory by the Government of India (4) Free of cost; Less likely to vaccinate: (1) Concerns about side effects (2) Vaccine conspiracy (3) Lack of confidence in vaccine effectiveness; Factors measured: Vaccine origin, receiving vaccine ASAP or waiting
15	Freeman, D. (2020) [51]	UK	Cross-sectional study	Adults over 18	To estimate willingness to receive COVID-19 vaccines and identify predictive socio-demographic factors and determine potential caues to guide information provision	71.70%	Factors measured: Age, gender, ethnicity, employment status, marital status, education, household income, housing situation, and region. Less likely to vaccinate: (1) Lower education (2) Black and mixed ethnicities (3) Not being single or widowed (4) Not being a homeowner (5) Having a child at school (6) Not being employed full-time (7) Not retired (8) Change in work	More likely to vaccinate: Help the community; Factors measured: Political beliefs	More likely to vaccinate: (1) Likely to be infected (2) Very high risk or moderate risk of severe COVID-19 illness; Less likely to vaccinate: (1) If speed of development would affect safety and efficacy (2) Degree to which receiving the vaccine may be physically unpleasant (3) Feeling experimented on (4) Anti-vaccination beliefs; Factors measured: Vaccine hesitancy, had COVID-19, had COVID-19 test, risk for COVID-19, adherence to government guidelines, conspiracy beliefs, rates of misinformation, explanatory factors for vaccine hesitancy
16	Salali, G.D. (2020) [52]	UK, Turkey	Cross-cultural study	Adults over 18	To examine levels of COVID-19 vaccine hesitancy and its association with beliefs about the origin of COVID-19	66% of participants in Turkey; 83% of participants in the UK	More likely to vaccinate: Men in Turkey; Less likely to vaccinate (in Turkey): (1) Having a graduate degree (2) Having children	More likely to vaccinate: Frequency of watching/listening/reading the news	More likely to vaccinate: (1) Believing in the natural origin of COVID-19 (2) Higher COVID-19-related anxiety scores
17	Al-Mohaithef, M. (2020) [25]	Saudi Arabia	Cross-sectional study	Adults over 18	To assess the prevalence of acceptance of COVID-19 vaccines and its determinants among people in Saudi Arabia	64.70%	Factors measured: Age, gender, marital status, nationality, city, profession, and education. More likely to vaccinate: (1) Older age group (above the age of 45) (2) Being married (3) Education level postgraduate degree or higher (4) Non-Saudi (5) Those working in the public sector	More likely to vaccinate: Trust the health system	More likely to vaccinate:High perceived risk of infection
18	Malik, A.A. (2020) [53]	USA	Cross-sectional study	18 years of age or older	To predict COVID-19 vaccine acceptance using demographic information and to identify the most vulnerable populations	67%	More likely to get vaccinated:Males, older adults, white people, Asian and Hispanic, those with higher educational level, retired and employed participants	More likely to get the vaccine: Those who trust in healthcare professionals, CDC, and local health departments	More likely to get the vaccine: Those who previous received influenza vaccines; Less likely to accept the vaccine: Regions with epicenters (geographic differences)
19	Kwok Ko (2020) [54]	Hong Kong	Cross-sectional online survey	Nurses	To estimate nurses’ influenza vaccination behaviors and intention to receive COVID-19 vaccines	63%	More likely to get vaccinated: Younger age	No social factors were recorded	More likely to get vaccinated: (1) Stronger vaccine confidence (2) More collective responsibility (3) Weaker complacency (4) Great stress work (5) Lack of personal protective equipment (6) Involvement in isolated rooms (7) Unfavorable attitudes towards workplace infection control policies
20	Kreps, Sarah (2020) [19]	USA	Cross-sectional study	Adults (30–58) years	To examine the factors associated with survey participants’ self-reported likelihood of selecting and receiving a hypothetical COVID-19 vaccine	79%	More likely intended to receive a vaccine: (1) People with educational attainment (2) Religious people; Less likely intended to receive a vaccine: (1) Women (2) Black people (3) Older adults	Political factors and democratic political partisanship were associated with preferences for choosing a hypothetical COVID-19 vaccine	More likely to get vaccinated: (1) High vaccine efficacy (2) Decrease in adverse effects in the vaccine (3) Long protection duration (4) Food and Drug Administration approval (5) National origin of vaccine (USA) and endorsements from CDC and WHO (6) Increased frequency of flu vaccination (7) Insured adults (8) Contact with COVID-19 cases
21	Leng A (2020) [55]	China	D-efficient discrete choice experiment	Adults over 18 years	To determine individual preferences for COVID-19 vaccinations in China, and to assess the factors influencing vaccination decision making to facilitate vaccination coverage	84.77%	Higher probability to vaccinate: (1) Older age individuals (2) Lower education level (3) Those with lower income	No social factors were recorded	Higher probability to vaccinate: (1) Higher trust in vaccines (2) High risk of infection (3) Vaccine effectiveness (4) Education in side effects (5) Higher proportion of acquaintances vaccinated (6) Vaccinations were free and voluntary (7) Smaller number of doses (8) Longer protection duration
22	Pogue, K. (2020) [56]	USA	Cross-sectional study	Adults	To understand the attitudes towards and obstacles facing vaccination with a potential COVID-19 vaccine.	68.57%	Income level and education level were all significantly correlated with intent to vaccinate.	Political ideology was significantly correlated with intent to vaccinate.	(1)Vaccine history (2) Longer testing (3) High vaccine efficacy (4) Location of vaccine development (United States) (5) Prior vaccine usage (6) The severity of COVID-19 and (7) Satisfaction with health insurance were all correlated with intent to vaccinate
23	Wang, K. (2020) [57]	Hong Kong, China	Cross-sectional study	Nurses	To examine the impact of the coronavirus disease 2019 (COVID-19) pandemic on changes in influenza vaccination acceptance, and identify factors associated with acceptance of potential COVID-19 vaccination	40.00%	Males and those who work in the private sector were found to be more likely to have acceptance of the vaccine	No social factors recorded	More likely to vaccinate: (1) Chronic conditions (2) Contact with suspected or confirmed COVID-19 patients (3) Accepted influenza vaccination; Less likely: (1) Efficacy, effectiveness, and safety of potential COVID-19 vaccines (2) Believing COVID-19 vaccination is unnecessary
24	Gagneux-Brunon A (2020) [40]	France	Cross-sectional study	Health workers	To determine COVID-19 vaccine acceptance rate in HCWs in France	76.90%	More likely to vaccinate: Older age, male gender; Less likely: Nurses and assistant nurses were less prone to accept the vaccine than physicians	No social factors recorded	More likely: (1) Fear about COVID-19 (2) Individual perceived risk (3) Previous flu vaccination; Less likely: Vaccine hesitancy
25	Detoc, (2020) [58]	France	Cross-sectional study	Random selection of the French adult general population and adult patients	To determine the proportion of people who intend to get vaccinated against COVID-19 in France or to participate in a vaccine clinical trial	77.60%	Age, gender, profession, and medical conditions were measured. Older individuals, males, and healthcare workers were more likely to vaccinate	No social factors recorded	(1) Fear about COVID-19 and individual perceived risk were associated with COVID-19 vaccine acceptance (2) Vaccine hesitancy was associated with a decrease in COVID-19 vaccine acceptance
26	Prati, G (2020) [20]	Italy	Cross-sectional study	Adults over 18 in Italy	To determine the extent to which Italian people intend to receive a vaccine against SARS-CoV-2, and to investigate its associations with worry, institutional trust, and beliefs about the non-natural origin of the virus	76%	Less likely: (1) Older people (2) Gender (3) Employment status and minority did not have an influence on intention to receive the vaccine.	Economic status did not have an influence on intention to receive the vaccine.	Less likely: (1) Lower levels of worry (2) Belief about the non-natural origin of the virus
27	Olagoke, Ayokunle A. (2020) [23]	USA	Cross-sectional study	Adults over 18	To examine the role of health locus of control (HLOC) in the relationship between religiosity and COVID-19 vaccination intention	The percentage was not measured	Gender, education, religion, ethnicity, employment, marital status, income, and medical conditions were measured. (1) Black/African American, unemployed/retired/disabled had lower COVID-19 vaccination intention (2) Negative association between religiosity and COVID-19 vaccination intention	No social factors measured	Personal belief against vaccines in general was indicative of lower COVID-19 vaccination intention
28	Palamenghi, L (2020) [59]	Italy	Cross-sectional study	Random selection of Adults representing the Italian population	To understand citizens’ perceptions about preventive behaviors, and their willingness to receive a vaccine for COVID-19	59%	Middle-aged individuals had lower willingness than individuals 18–34 years old and over 60 years old	Willingness to vaccinate correlated with beliefs and trust of scientific research	(1) Willingness to vaccinate was correlated with general attitude towards vaccines’ efficacy (2) No significant difference between smokers’ and non-smokers’ willingness to vaccinate against COVID-19
29	Bell, S. (2020) [60]	UK	An online cross-sectional survey and semi-structured interviews	Parents and guardians	To investigate parents’ and guardians’ views on the acceptability of a future COVID-19 vaccine	Parents for themselves (Definitely 55.8%; Unsure but leaning towards yes 34.3%), for their children (Definitely 48.2%; Unsure but leaning towards yes 40.9%)	Black, Asian, Chinese, Mixed, and lower income households were more likely to reject a COVID-19 vaccine for themselves and their children	No social factors measured	More likely to vaccinate: (1) Self-protection from COVID-19 (2) COVID-19 vaccine safety and effectiveness (3) Rapid development of the vaccine.
30	Borriello A (2020) [61]	Australia	Cross-sectional study	Australian residents	To investigate the vaccine characteristics that matter the most to Australian citizens, and to explore the potential uptake of a COVID-19 vaccine in Australia	Average = 86.03%	Age, being female, being single, and income level were associated with intention to get the vaccine	No social factors measured	More likely: (1) Vaccine availability in a shorter time (2) Less severe side effects (3) Vaccine effectiveness (4) Vaccine price
31	Faasse, K (2020) [62]	Australia	Cross-sectional study	Australian residents	To assess uncertainty and misconceptions about COVID-19	Definitely would: 60.5%;Probably would: 20.6%	Age, gender, state, ethnicity, and education were measured.Males and older individuals were associated with intending to get the vaccine	No social factors recorded	More likely: (1) Received a seasonal flu vaccine (2) Increased exposure to media coverage (3) Worry or concern about the outbreak (4) Greater understanding of the virus (5) Confidence in government information (6) Vaccine effectiveness
32	Dong, Dong (2020) [63]	China	A discrete choice experiment	General population	To examine how factors related to vaccine characteristics, their social normative influence, and convenience of vaccination can affect the public’s preference for the uptake of COVID-19 vaccines in China	Approximately: 78%	More likely: (1) Women, (2) Had children (3) Lived in an urban area	No social factors recorded	More likely: (1) High effectiveness of the vaccine (2) Long protective duration (3) Few adverse effects (4) Place of manufacturing (5) COVID-19 vaccine’s price (6) Number of injections (7) Vaccinated in the past
33	Graffigna, G (2020) [64]	Italy	Cross-sectional study	Random selection of Adults (over 18)	To understand how adult citizens’ health engagement, perceived COVID-19 susceptibility and severity, and general vaccine-related attitudes affect the willingness to vaccinate against COVID-19	58.60%	More likely: Older individuals have more vaccine uptake, based on their correlating health engagement	No social factors measured	More likely: (1) Higher ratings of health engagement (2) High perceived susceptibility towards COVID-19 and disease severity
34	Roozenbeek J (2020) [65]	Ireland, USA, Spain, Mexico, UK	Cross-sectional study	Adults	To explore whether susceptibility to misinformation is a significant predictor of compliance with health guidance measures	The percentage was not measured	Age, gender, education, and minority status were measured; More likely: Being older and male	Political beliefs: No differences found between different political ideology with regards to the intention to use COVID-19 vaccine; More likely to vaccinate: higher trust in scientists	Less likely to vaccinate: Conspiracy beliefs and Misinformation
35	Grüner, S. (2020) [26]	Germany	Cross-sectional study	Students with and without healthcare background, and healthcare professionals	To better understand which determinants can explain the willingness to get vaccinated against COVID-19	68%	The willingness to be vaccinated against COVID-19 is quite similar among age groups and gender; More likely to vaccinate: Healthcare professionals, healthcare and non-healthcare students	More likely to vaccinate: Trust in the mass media, government, and the healthcare system	More likely: (1) Immunocompromised person/individuals with family members who have compromised immune systems (2) Those who think deliberately; Less likely: (1) Good health status (2) Those who use homoeopathy or naturopathy
36	Wong, L.P. (2020) [66]	Malaysia	Cross-sectional study	Malaysian residents (18–70 years of age)	To identify predictors of participants’ intention to receive a COVID-19 vaccine, and their WTP for COVID-19 vaccination	48.2% (will be taking it); 29.8% (probably will be taking it)	More Likely: Males, those with highest education level, and those who are retired or unemployed. No differences found between age, ethnicity, living area, income level	More likely to vaccinate: Housewife	More likely: (1) Contact with people infected by COVID-19 (2) High risk of getting COVID-19 (3) Fear about getting COVID-19 (4) Vaccine confidence (5) Recommended by people, family; Less likely: (1) Adverse side effects (2) Vaccine efficacy (3) Vaccine safety (4) Personal beliefs against vaccine (5) Vaccine price (6) Inadequate information
37	Bokemper SE (2020) [67]	USA	Randomized control experiment	Random selection of Adults (over 18)	To examine how timing and elite endorsement effect public opinion about a COVID-19 vaccine	51%	Higher vaccine acceptance amongst Democrats than Republicans.	Political beliefs and endorsement by public figures influence COVID-19 vaccine approval, confidence, and uptake; More likely to take the vaccine: (1) If approved after the election, rather than one week prior (2) Individuals who supported Dr. Fauci	No contextual factors were recorded
38	Williams, Lynn (2020) [68]	UK	Cross-sectional study (questionnaire and free-response questions)	Older adults (aged over 65) and those with chronic respiratory disease (aged 18–64) (asthma or COPD)	To understand the barriers and facilitators to receiving a future COVID-19 vaccine	86% (58% definitely, 27% probably)	There were no differences in willingness to have the vaccine based on age group or gender	There were no differences in willingness to have the vaccine based on socioeconomic status; Less likely to vaccinate: Trust in media	More likely: (1) Belief that COVID-19 outbreak will continue for a long time (2) Personal health (3) Severity of COVID-19 disease (4) Health consequences to others; Less likely: Concerns about vaccine safety
39	Lin, Y. (2020) [69]	China	Cross-sectional study	Chinese citizens at least 18 years old	To understand coronavirus disease 2019 (COVID-19) vaccine demand and hesitancy by assessing the public’s vaccination intention and willingness to pay (WTP)	83.3% (28.7%, 54.6% probably)	Age, gender, marital status, education level, income level, and location were measured. Strong correlation with definite intention to vaccinate: Central and southern regions	No social factors recorded	Measured factors: Past experience with COVID-19, health history, worry about getting COVID-19, perceived benefits, perceived barriers. More likely to get the vaccine: (1) Good overall health (2) Fear about COVID-19 (3) Vaccine confidence (4) Recommendation by general population (5) Preference for domestically made COVID-19 vaccine rather than foreign-made; Less likely: Concerns about the safety, efficacy, or side effects of the vaccine
40	Grech, V (2020) [22]	Malta	Cross-sectional study	Healthcare workers	To ascertain Maltese healthcare workers’ hesitancy to a novel COVID-19 vaccine, and correlate this with influenza vaccine uptake	52%	Males and doctors were likelier to takethe vaccine.	No social factors recorded	More likely: Likelihood of influenza vaccination. Concerns raised were related to insufficient knowledge about such a novel vaccine, and long-term side effects.
41	Barello, S (2020) [70]	Italy	Cross-sectional study	University Students	To explore university students’ attitudes towards a future vaccine to prevent COVID-19, and to evaluate the impact of the university curricula on the intention to vaccinate.	86.10%	Type of education studied and level of study measured.There were no significant differences between healthcare students and non-healthcare students with regards to intention to vaccinate	Students’ intentions to vaccinate did not significantly differ based on social characteristics	No specific factors
42	Grech, V (2020) [71]	Malta	Cross-sectional study	General practitioners and trainees	To ascertain the degree of vaccine hesitancy of GPs and GP trainees in Malta with regrd to influenza vaccination and novel COVID-19 vaccine.	70.8% of GPs, 29.6% Trainees	More likely to take vaccine (1) Increasing age (2) General practitioners	No social factors recorded	Likelihood of taking COVID-19 vaccine correlated with (1) Taking influenza vaccine (2) Vaccine effectiveness (3) Vaccine side effects (4) Anti-vaccines beliefs
43	Grech, V. (2020) [72]	Malta	Cross-sectional study	University students, academics, and administrators	To ascertain degree of vaccine hesitancy with regard to influenza and COVID-19 vaccination.	31%	More likely to vaccinate: (1) Academics, followed by students and support staff (2) Faculty: Medicine (3) Older age (4) Males	No social factors recorded	Proportion of those likely to take the COVID-19 vaccine was directly related to: (1) Likelihood of taking influenza vaccine (2) Vaccine effectiveness (3) Vaccine side effects (4) General opposition to vaccines
44	Kose, S (2020) [24]	Turkey	Cross-sectional study	Healthcare workers	To determine the acceptance status of COVID-19 vaccines amongst healthcare professionals	68.60%	Sex, age, occupation, smoking, and living place were measured. Men and healthcare workers were more likely to take the vaccines	No social factors recorded	Availability of vaccine, efficacy of vaccine, and previous vaccination were measured. People who were previously vaccinated were more likely to take the vaccines
45	Dror, A (2020) [9]	Israel	Cross-sectional study	Adults over 18	To evaluate current vaccination compliance rates among the Israeli population	75%	Age, region, profession, gender, marital status, and parenthood were measured. More likely to vaccinate: (1) Males (2) Doctors more likely than nurses (3) Internal medicine doctors more likely than general surgery doctors (4) Individuals who lost their jobs more likely than frontline workers	No social factors recorded	More likely to vaccinate: (1) People vaccinated against seasonal influenza (2) High vaccine safety (3) Rapid vaccine development (4) Fewer potential side effects (5) Associated COVID-19 illness (6) Contact with COVID-19-positive patients (7) At high risk to be infected with COVID-19
46	Akarsu, B (2020) [73]	Turkey	Cross-sectional study	Adults over 18 that use social media or smartphones	To investigate the thoughts and attitudes of individuals towards a future COVID-19 vaccine	49.70%	Gender, age, and occupation were measured. More likely: (1) sSudents (2) Women (3) Higher education levels (4) Healthcare workers	No social factors recorded	Increasing anxiety, private insurance, and regular flu shots were correlated with increase vaccine uptake
47	Marcec, R (2020) [74]	European Countries	Cross-sectional study	general population	To assess public opinion about attitudes towards SARS-CoV-2 vaccination in 26 European countries.	58%	Demographic factors were not recorded	Social factors were not recorded	Public perceptions on vaccine uptake and hesitancy were measured
48	Guidry, P (2020) [75]	USA	Cross-sectional study	Adults over 18	To assess psychosocial predictors of U.S. adults’ willingness to get a future COVID-19 vaccine	30.7% (definitely) 29.2% (probably)	Age, gender, religion, ethnicity, and education were measured.More likely to increase vaccine uptake: (1) Participants with higher education (2) White people (3) Younger people	Participants with higher socio-economic status were more likely to vaccinate	(1) Rushed vaccine development reduced intention to take it (2) Insurance coverage made vaccine uptake more likely

**Table 2:** All factors derived from the literature.

Theoretical Constructs	Reference of the Study
(1) Demographics	
Age	[9,19,20,21,22,24,25,26,27,28,37,38,39,40,43,45,46,47,49,50,51,53,54,55,57,58,59,61,62,64,65,66,68,69,71,72,73,75] *
Gender	[9,19,20,21,22,23,24,25,26,27,28,37,38,39,40,43,44,45,46,47,49,50,51,52,53,61,62,63,65,66,68,69,72,73,75] *
Education level	[9,19,21,23,24,25,27,28,39,43,44,45,46,47,50,51,52,53,55,56,58,62,63,65,66,69,70,72,73,75] *
Income level	[21,23,28,39,43,45,46,50,51,55,56,60,61,63,66,69,75] *
Employment status	[20,21,23,28,43,44,46,47,51,53,58,61,63,66,69,73] *
Socio-geographic (e.g., rural vs. urban)	[9,21,24,25,27,28,39,45,46,47,51,52,53,55,62,63,66,69] *
Race/ethnicity	[21,23,25,28,39,43,44,45,50,51,53,60,62,65,66,75] *
Having children	[9,51,52,63] *
Religiosity	[19,23,28,39,44,45,50,51] *
Marital status	[9,21,23,25,28,38,43,45,46,51,61,63,69] *
Profession	[9,22,24,25,27,28,37,38,40,43,58,71,72,73] *
Place of work	[53,54,57] *
Smoking status	[24,26,59] *
(2) Social factors	
Social solidarity	[44,51]
Socioeconomic status	[20,37,50,68] *
Social density and prosocial concern	[48,51] *
Child protection/parental concerns	[47,52,60] *
Communication and media	[26,44,62,68] *
Trust in government	35, [26,53,62,67] *
Political beliefs	[19,43,44,45,49,50,51,56,65,67] *
Recent or upcoming travel	[45]
Trust in scientists/WHO/CDC/FDA	[19,25,26,39,50,51,53,55,59,60,65] *
Work stress/anxiety	[52,54,73] *
(3) Vaccination beliefs and attitudes	
Believing COVID-19 vaccination is unnecessary	[57] *
Vaccine hesitancy	[20,21,24,40,43,44,51,52,58] *
Personal belief against vaccines	[20,23,66] *
Preference for natural immunity	[44] *
Anti-vaccine attitudes or beliefs	[21,51,71,72] *
Conspiracy beliefs	[20,27,49,51,65] *
Vaccine confidence	[24,27,51,54,55,60,65,66,69] *
(4) Vaccine-related perceptions	
Vaccine efficacy/effectiveness	[9,19,21,24,27,28,39,44,45,46,47,50,51,55,56,57,59,60,61,62,63,66,69,71,72,73,75] *
Vaccine safety	[9,21,27,39,44,46,47,50,51,57,60,61,66,67,68,69] *
Vaccine price	[27,45,46,47,55,61,63,66] *
Rapid development of the vaccine	[9,44,51,60,61] *
Vaccine country origin/national	[19,27,46,56,63,69] *
Vaccine adverse side effect	[9,19,22,27,39,44,45,55,61,63,66,69,71,72] *
Frequency of injection	[46,55,63] *
Long protection duration	[19,45,55,63] *
Influenza vaccination history	[9,19,21,22,37,39,40,46,47,53,56,57,62,63,71,72] *
(5) Health-related perceptions	
Health history/medical conditions	[23,26,43,45,53,57,58,68,69] *
High risk of COVID-19 infection	[9,25,28,39,40,45,46,51,52,55,60,64,66] *
Being sick with COVID-19	[39,45,50,51,69] *
Insurance coverage	[19,45,50,56,63,73,75] *
Family history of compromised immune systems	[26,39] *
Contact with COVID-19 cases	[9,19,39,46,51,53,57,66] *
Trust in homeopathy and naturopathy	[26] *
Fear about COVID-19 (Self-protection)	[26,40,43,52,56,58,60,62,64,66,68,69] *
(6) Perceived barriers	
Financial barriers	[50] *
Lack of trust	[20,21,51] *
Misinformation	[22,51,65,66,71,72] *
Concerns about commercial profiteering	[44] *
(7) Vaccine recommendations	
Vaccine recommendation by health professional/scientists	[27,44,45,46,60] *
Vaccine recommendation by friends or family	[55,60,66,69] *
Government advice to vaccinate	[44,67] *
Mandatory by the government	[27,44] *

(*) means the factor is significantly associated with intention to use COVID-19.

## Appendix A

The search strategy was done using four databases:

### Appendix A.1. PubMed Search Strategy

**Table A1:** PubMed search strategy.

Concepts	Vaccine	Intention	Covid-19
Keywords (title, abstract, author keywords)	Vaccin* OR Immuniz* OR Immunis* OR Inoculat* OR Moderna OR Pfizer OR anti-vaccin*	Hesitan* OR accept* OR Refus* OR preference* OR Willingness OR Intention OR trust OR mistrust OR attitude* OR avoidance OR distrust OR knowledge OR doubt OR fear OR perception* OR misconception* OR misinformation OR belief OR dilemma OR behavio* OR concern* OR delay OR confidence OR adherence OR nonadherence OR noncomplian* OR complian* OR uptake OR opinion* OR anxiety* OR decision∗	CADTH search
MeSH Terms (PubMed)	Immunization OR Vaccines OR Mass Vaccination	Attitude to health OR Health attitudes OR Vaccination Refusal OR Health Knowledge, Attitudes, Practice OR Patient Acceptance of Health Care OR trust	

#### Appendix A.1.1. CADTH Search

((Coronavirus[mh:noexp] OR Betacoronavirus[mh:noexp] OR Coronavirus Infections[mh:noexp]) AND (Disease Outbreaks[mh:noexp] OR Epidemics[mh:noexp] OR Pandemics[mh])) OR COVID-19 diagnostic testing [Supplementary Concept] OR COVID-19 drug treatment [Supplementary Concept] OR COVID-19 serotherapy [Supplementary Concept] OR COVID-19 vaccine [Supplementary Concept] OR spike glycoprotein, COVID-19 virus [Supplementary Concept] OR COVID-19 [Supplementary Concept] OR severe acute respiratory syndrome coronavirus 2 [Supplementary Concept] OR nCoV[tiab] OR nCoV[tt] OR 2019nCoV[tiab] OR 2019nCoV[tt] OR 19nCoV[tiab] OR 19nCoV[tt] OR COVID19*[tiab] OR COVID19*[tt] OR COVID[tiab] OR COVID[tt] OR SARS-CoV-2[tiab] OR SARS-CoV-2[tt] OR SARSCOV-2[tiab] OR SARSCOV-2[tt] OR SARSCOV2[tiab] OR SARSCOV2[tt] OR Severe Acute Respiratory Syndrome Coronavirus 2[tiab] OR Severe Acute Respiratory Syndrome Coronavirus 2[tt] OR ((severe acute respiratory syndrome[tiab] OR severe acute respiratory syndrome[tt]) AND (corona virus 2[tiab] OR corona virus 2[tt])) OR new coronavirus[tiab] OR (new[tt] AND coronavirus[tt]) OR novel coronavirus[tiab] OR novel coronavirus[tt] OR novel corona virus[tiab] OR (novel[tt] AND corona virus[tt]) OR novel CoV[tiab] OR (novel[tt] AND CoV[tt]) OR novel HCoV[tiab] OR (novel[tt] AND HCoV[tt]) OR ((“19″[tiab] OR “19”[tt] OR “2019”[tiab] OR “2019”[tt] OR Wuhan[tiab] OR Wuhan[tt] OR Hubei[tiab] OR Hubei[tt]) AND (coronavirus*[tiab] OR coronavirus*[tt] OR corona virus*[tiab] OR corona virus*[tt] OR CoV[tiab] OR CoV[tt] OR HCoV[tiab] OR HCoV[tt])) OR ((coronavirus*[tiab] OR coronavirus*[tt] OR corona virus*[tiab] OR corona virus*[tt] OR betacoronavirus*[tiab] OR betacoronavirus*[tt]) AND (outbreak*[tiab] OR outbreak*[tt] OR epidemic*[tiab] OR epidemic*[tt] OR pandemic*[tiab] OR pandemic*[tt] OR crisis[tiab] OR crisis[tt])) OR ((Wuhan[tiab] OR Wuhan[tt] OR Hubei[tiab] OR Hubei[tt]) AND (pneumonia[tiab] OR pneumonia[tt])).

#### Appendix A.1.2. Search Strings

#1: (Vaccin*[Text Word] OR Immuniz*[Text Word] OR Immunis*[Text Word] OR Inoculat*[Text Word] OR Moderna[Text Word] OR Pfizer[Text Word] OR anti-vaccin*[Text Word]) OR (Immunization[MeSH Terms] OR Vaccines[MeSH Terms] OR Mass Vaccination[MeSH Terms])= 617,425 results.

#2: (Hesitan*[Text Word] OR accept*[Text Word] OR Refus*[Text Word] OR preference*[Text Word] OR Willingness[Text Word] OR Intention[Text Word] OR trust[Text Word] OR mistrust[Text Word] OR attitude*[Text Word] OR avoidance[Text Word] OR distrust[Text Word] OR knowledge[Text Word] OR doubt[Text Word] OR fear[Text Word] OR perception*[Text Word] OR misconception*[Text Word] OR misinformation[Text Word] OR belief[Text Word] OR dilemma[Text Word] OR behavio*[Text Word] OR concern*[Text Word] OR delay[Text Word] OR confidence[Text Word] OR adherence[Text Word] OR nonadherence[Text Word] OR noncomplian*[Text Word] OR complian*[Text Word] OR uptake[Text Word] OR opinion*[Text Word] OR anxiety*[Text Word] OR decision∗[Text Word]) OR (Attitude to health[MeSH Terms] OR Health attitudes[MeSH Terms] OR Vaccination Refusal[MeSH Terms] OR Health Knowledge, Attitudes, Practice[MeSH Terms] OR Patient Acceptance of Health Care[MeSH Terms] OR trust[MeSH Terms])= 5,668,494 results.

#3: ((Coronavirus[mh:noexp] OR Betacoronavirus[mh:noexp] OR Coronavirus Infections[mh:noexp]) AND (Disease Outbreaks[mh:noexp] OR Epidemics[mh:noexp] OR Pandemics[mh])) OR COVID-19 diagnostic testing [Supplementary Concept] OR COVID-19 drug treatment [Supplementary Concept] OR COVID-19 serotherapy [Supplementary Concept] OR COVID-19 vaccine [Supplementary Concept] OR spike glycoprotein, COVID-19 virus [Supplementary Concept] OR COVID-19 [Supplementary Concept] OR severe acute respiratory syndrome coronavirus 2 [Supplementary Concept] OR nCoV[tiab] OR nCoV[tt] OR 2019nCoV[tiab] OR 2019nCoV[tt] OR 19nCoV[tiab] OR 19nCoV[tt] OR COVID19*[tiab] OR COVID19*[tt] OR COVID[tiab] OR COVID[tt] OR SARS-CoV-2[tiab] OR SARS-CoV-2[tt] OR SARSCOV-2[tiab] OR SARSCOV-2[tt] OR SARSCOV2[tiab] OR SARSCOV2[tt] OR Severe Acute Respiratory Syndrome Coronavirus 2[tiab] OR Severe Acute Respiratory Syndrome Coronavirus 2[tt] OR ((severe acute respiratory syndrome[tiab] OR severe acute respiratory syndrome[tt]) AND (corona virus 2[tiab] OR corona virus 2[tt])) OR new coronavirus[tiab] OR (new[tt] AND coronavirus[tt]) OR novel coronavirus[tiab] OR novel coronavirus[tt] OR novel corona virus[tiab] OR (novel[tt] AND corona virus[tt]) OR novel CoV[tiab] OR (novel[tt] AND CoV[tt]) OR novel HCoV[tiab] OR (novel[tt] AND HCoV[tt]) OR ((“19″[tiab] OR “19”[tt] OR “2019”[tiab] OR “2019”[tt] OR Wuhan[tiab] OR Wuhan[tt] OR Hubei[tiab] OR Hubei[tt]) AND (coronavirus*[tiab] OR coronavirus*[tt] OR corona virus*[tiab] OR corona virus*[tt] OR CoV[tiab] OR CoV[tt] OR HCoV[tiab] OR HCoV[tt])) OR ((coronavirus*[tiab] OR coronavirus*[tt] OR corona virus*[tiab] OR corona virus*[tt] OR betacoronavirus*[tiab] OR betacoronavirus*[tt]) AND (outbreak*[tiab] OR outbreak*[tt] OR epidemic*[tiab] OR epidemic*[tt] OR pandemic*[tiab] OR pandemic*[tt] OR crisis[tiab] OR crisis[tt])) OR ((Wuhan[tiab] OR Wuhan[tt] OR Hubei[tiab] OR Hubei[tt]) AND (pneumonia[tiab] OR pneumonia[tt]))= 97,841 results.

#4: (“2019/11/1”[Date - Publication] : “2020/12/25”[Date - Publication]= 1,806,287 results.

Final Search #5= #1 AND #2 AND #3 AND #4= 1,452 results.

((Vaccin*[Text Word] OR Immuniz*[Text Word] OR Immunis*[Text Word] OR Inoculat*[Text Word] OR Moderna[Text Word] OR Pfizer[Text Word] OR anti-vaccin*[Text Word]) OR (Immunization[MeSH Terms] OR Vaccines[MeSH Terms] OR Mass Vaccination[MeSH Terms])) AND ((Hesitan*[Text Word] OR accept*[Text Word] OR Refus*[Text Word] OR preference*[Text Word] OR Willingness[Text Word] OR Intention[Text Word] OR trust[Text Word] OR mistrust[Text Word] OR attitude*[Text Word] OR avoidance[Text Word] OR distrust[Text Word] OR knowledge[Text Word] OR doubt[Text Word] OR fear[Text Word] OR perception*[Text Word] OR misconception*[Text Word] OR misinformation[Text Word] OR belief[Text Word] OR dilemma[Text Word] OR behavio*[Text Word] OR concern*[Text Word] OR delay[Text Word] OR confidence[Text Word] OR adherence[Text Word] OR nonadherence[Text Word] OR noncomplian*[Text Word] OR complian*[Text Word] OR uptake[Text Word] OR opinion*[Text Word] OR anxiety*[Text Word] OR decision∗[Text Word]) OR (Attitude to health[MeSH Terms] OR Health attitudes[MeSH Terms] OR Vaccination Refusal[MeSH Terms] OR Health Knowledge, Attitudes, Practice[MeSH Terms] OR Patient Acceptance of Health Care[MeSH Terms] OR trust[MeSH Terms])) AND (((Coronavirus[mh:noexp] OR Betacoronavirus[mh:noexp] OR Coronavirus Infections[mh:noexp]) AND (Disease Outbreaks[mh:noexp] OR Epidemics[mh:noexp] OR Pandemics[mh])) OR COVID-19 diagnostic testing [Supplementary Concept] OR COVID-19 drug treatment [Supplementary Concept] OR COVID-19 serotherapy [Supplementary Concept] OR COVID-19 vaccine [Supplementary Concept] OR spike glycoprotein, COVID-19 virus [Supplementary Concept] OR COVID-19 [Supplementary Concept] OR severe acute respiratory syndrome coronavirus 2 [Supplementary Concept] OR nCoV[tiab] OR nCoV[tt] OR 2019nCoV[tiab] OR 2019nCoV[tt] OR 19nCoV[tiab] OR 19nCoV[tt] OR COVID19*[tiab] OR COVID19*[tt] OR COVID[tiab] OR COVID[tt] OR SARS-CoV-2[tiab] OR SARS-CoV-2[tt] OR SARSCOV-2[tiab] OR SARSCOV-2[tt] OR SARSCOV2[tiab] OR SARSCOV2[tt] OR Severe Acute Respiratory Syndrome Coronavirus 2[tiab] OR Severe Acute Respiratory Syndrome Coronavirus 2[tt] OR ((severe acute respiratory syndrome[tiab] OR severe acute respiratory syndrome[tt]) AND (corona virus 2[tiab] OR corona virus 2[tt])) OR new coronavirus[tiab] OR (new[tt] AND coronavirus[tt]) OR novel coronavirus[tiab] OR novel coronavirus[tt] OR novel corona virus[tiab] OR (novel[tt] AND corona virus[tt]) OR novel CoV[tiab] OR (novel[tt] AND CoV[tt]) OR novel HCoV[tiab] OR (novel[tt] AND.

HCoV[tt]) OR ((“19″[tiab] OR “19”[tt] OR “2019”[tiab] OR “2019”[tt] OR Wuhan[tiab] OR Wuhan[tt] OR Hubei[tiab] OR Hubei[tt]) AND (coronavirus*[tiab] OR coronavirus*[tt] OR corona virus*[tiab] OR corona virus*[tt] OR CoV[tiab] OR CoV[tt] OR HCoV[tiab] OR HCoV[tt])) OR ((coronavirus*[tiab] OR coronavirus*[tt] OR corona virus*[tiab] OR corona virus*[tt] OR betacoronavirus*[tiab] OR betacorona.

virus*[tt]) AND (outbreak*[tiab] OR outbreak*[tt] OR epidemic*[tiab] OR epidemic*[tt] OR pandemic*[tiab] OR pandemic*[tt] OR crisis[tiab] OR crisis[tt])) OR ((Wuhan[tiab] OR Wuhan[tt] OR Hubei[tiab] OR Hubei[tt]) AND (pneumonia[tiab] OR pneumonia[tt]))) AND ((“2019/11/1”[Date - Publication]: “2020/12/25”[Date - Publication])).

### Appendix A.2. Scopus Search Strategy

#### Search Method (SCOPUS)

27 January 2021 (2002 results).

**Table A2:** Scopus search strategy.

Concepts	Vaccine	Intention	Covid-19
Keywords (title, abstract, author keywords)	Vaccine Vaccination Immunization Inoculation Inoculate Immunize Anti-vaccination Moderna Pfizer	hesitan* OR accept* OR refus* OR preference* OR willingness OR intention OR trust OR mistrust OR attitude* OR avoidance OR distrust OR knowledge OR doubt OR fear OR perception* OR misconception* OR misinformation OR belief OR dilemma OR behavio* OR concern* OR delay OR confidence OR adherence OR nonadherence OR noncomplian* OR complian* OR uptake OR opinion* OR anxiety* OR decision^∗^ OR “public opinion” OR “health attitudes”	CADTH search

TITLE-ABS-KEY (vaccin* OR immunis* OR immuniz* OR inocula* OR “anti-vaccin*” OR moderna OR pfizer) AND TITLE-ABS-KEY (hesitan* OR accept* OR refus* OR preference* OR willingness OR intention OR trust OR mistrust OR attitude* OR avoidance OR distrust OR knowledge OR doubt OR fear OR perception* OR misconception* OR misinformation OR belief OR dilemma OR behavio* OR concern* OR delay OR confidence OR adherence OR nonadherence OR noncomplian* OR complian* OR uptake OR opinion* OR anxiety* OR decision* OR “public AND opinion” OR “health AND attitudes”) AND ((KEY (coronavirus OR betacoronavirus OR “coronavirus infections”) AND KEY (“disease outbreaks” OR epidemics OR pandemics)) OR (TITLE-ABS-KEY (ncov* OR 2019ncov OR 19ncov OR covid19* OR covid OR sars-cov-2 OR sars-cov2 OR sarscov-2 OR sarscov2 OR “Severe Acute Respiratory Syndrome Coronavirus 2” OR “Severe Acute Respiratory Syndrome Corona Virus 2”)) OR (TITLE-ABS-KEY ((new W/3 coronavirus*) OR (new W/3 “corona virus*”) OR (new W/3 betacoronavirus*) OR (new W/3 cov) OR (new W/3 hcov) OR (novel W/3 coronavirus*) OR (novel W/3 “corona virus*”) OR (novel W/3 betacoronavirus*) OR (novel W/3 cov) OR (novel W/3 hcov) OR (19 W/3 coronavirus*) OR (19 W/3 “corona virus*”) OR (19 W/3 betacoronavirus*) OR (19 W/3 cov) OR (19 W/3 hcov) OR (2019 W/3 coronavirus*) OR (2019 W/3 “corona virus*”) OR (2019 W/3 betacoronavirus*) OR (2019 W/3 cov) OR (2019 W/3 hcov) OR (wuhan W/3 coronavirus*) OR (wuhan W/3 “corona virus*”) OR (wuhan W/3 betacoronavirus*) OR (wuhan W/3 cov) OR (wuhan W/3 hcov) OR (hubei W/3 coronavirus*) OR (hubei W/3 “corona virus*”) OR (hubei W/3 betacoronavirus*) OR (hubei W/3 cov) OR (hubei W/3 hcov) OR (china W/3 coronavirus*) OR (china W/3 “corona virus*”) OR (china W/3 betacoronavirus*) OR (china W/3 cov) OR (china W/3 hcov) OR (chinese W/3 coronavirus*) OR (chinese W/3 “corona virus*”) OR (chinese W/3 betacoronavirus*) OR (chinese W/3 cov) OR (chinese W/3 hcov))) OR (TITLE-ABS-KEY ((coronavirus* W/3 pandemic*) OR (coronavirus* W/3 epidemic*) OR (coronavirus* W/3 outbreak*) OR (coronavirus* W/3 crisis) OR (“corona virus*” W/3 pandemic*) OR (“corona virus*” W/3 epidemic*) OR (“corona virus*” W/3 outbreak*) OR (“corona virus*” W/3 crisis) OR (betacoronavirus* W/3 pandemic*) OR (betacoronavirus* W/3 epidemic*) OR (betacoronavirus* W/3 outbreak*) OR (betacoronavirus* W/3 crisis))) OR (TITLE-ABS-KEY ((wuhan W/5 pneumonia) OR (hubei W/5 pneumonia)))) AND (PUBYEAR > 2018) AND (LIMIT-TO (PUBYEAR , 2020) OR LIMIT-TO (PUBYEAR , 2019)).

### Appendix A.3. PsycINFO Search Strategy

#### Search Method (PsycInfo)

27 January 2021 (61 results).

**Table A3:** PsycINFO search strategy.

Concepts	Vaccine	Intention	Covid-19
Keywords(title, abstract, author keywords)	Vaccine Vaccination Immunization Inoculation Inoculate Immunize Anti-vaccination Moderna Pfizer	hesitan* OR accept* OR refus* OR preference* OR willingness OR intention OR trust OR mistrust OR attitude* OR avoidance OR distrust OR knowledge OR doubt OR fear OR perception* OR misconception* OR misinformation OR belief OR dilemma OR behavio* OR concern* OR delay OR confidence OR adherence OR nonadherence OR noncomplian* OR complian* OR uptake OR opinion* OR anxiety* OR decision∗	Coronavirus COVID-19 SARS-CoV-2 COVID19 2019-ncov Cov19
Index Terms (PsycInfo)	Immunization	Health Attitudes Public Opinion	Coronavirus Disease outbreaks

Any Field: vaccin* *OR* Any Field: immunis* *OR* Any Field: immuniz* *OR* Any Field: inocula* *OR* Any Field: “anti-vaccin*” *OR* Any Field: moderna *OR* Any Field: pfizer *OR* Any Field: immunization *AND* Any Field: hesitan* *OR* Any Field: accept* *OR* Any Field: refus* *OR* Any Field: preference* *OR* Any Field: willingness *OR* Any Field: intention *OR* Any Field: trust *OR* Any Field: mistrust *OR* Any Field: attitude* *OR* Any Field: avoidance *OR* Any Field: distrust *OR* Any Field: knowledge *OR* Any Field: doubt *OR* Any Field: fear *OR* Any Field: perception* *OR* Any Field: misconception* *OR* Any Field: misinformation *OR* Any Field: belief *OR* Any Field: dilemma *OR* Any Field: behavio* *OR* Any Field: concern* *OR* Any Field: delay *OR* Any Field: confidence *OR* Any Field: adherence *OR* Any Field: nonadherence *OR* Any Field: noncomplian* *OR* Any Field: complian* *OR* Any Field: uptake *OR* Any Field: opinion* *OR* Any Field: anxiety* *OR* Any Field: decision* *OR* Any Field: “health attitudes” *OR* Any Field: “public opinion” *AND* Any Field: coronavirus *OR* Any Field: “COVID-19” *OR* Any Field: “SARS-CoV-2” *OR* Any Field: COVID19 *OR* Any Field: “2019-ncov” *OR* Any Field: cov19 *OR* Any Field: “disease outbreaks” *AND* Year: 2019 *To* 2020

### Appendix A.4. CINAHL Search Strategy

**Table A4:** CINAHL search strategy.

Concepts	Vaccine	Intention	Covid-19
Keywords(title, abstract, author, keywords)	Vaccine Vaccination Immunization Inoculation Inoculate Immunize Moderna Pfizer	hesitan* OR accept* OR refus* OR preference* OR willingness OR intention OR trust OR mistrust OR attitude* OR avoidance OR distrust OR knowledge OR doubt OR fear OR perception* OR misconception* OR misinformation OR belief OR dilemma OR behavio* OR concern* OR delay OR confidence OR adherence OR nonadherence OR noncomplian* OR complian* OR uptake OR opinion* OR anxiety* OR decision^∗^	Coronavirus COVID-19 SARS-CoV-2 Vaccine COVID19 2019-ncov Cov19
CINAHL/MeSH Subject Headings	Immunization Vaccines COVID-19 Vaccines	Attitude to Vaccines Public Opinion Health Beliefs Trust Worry	COVID-19 Disease Outbreaks

#### Search Method

27 January 2021

**Limiters**—Published Date: 20191101-20201231

1-S1 (97,435 results):

TX (vaccin* OR immunis* OR immuniz* OR inoculat* OR “anti-vaccin*” OR Moderna OR Pfizer OR immunization OR vaccines OR “COVID-19 Vaccines”).

2-S2 (2,169,483) results:

TX (hesitan* OR accept* OR refus* OR willing* OR intent* OR prefer* OR avoid* OR attitude* OR trust OR knowledge OR confidence OR opinion OR decision* OR perception OR perceive* OR concern* OR belie* OR adhere* OR nonadhere* OR complian* OR noncomplian* OR mistrust OR doubt* OR fear* OR misconception* OR misinform* OR dilemma OR behav* OR uptake OR anxiet* OR delay OR “attitude to vaccines” OR “public opinion” OR “health beliefs” OR worry).

3-S3 (66,102 results):

TX (“COVID-19” OR coronavirus OR “SARS-Cov-2” OR COVID19 OR “2019-ncov” OR cov19 OR “disease outbreaks”).

4-S4 (721 results):

S1 AND S2 AND S3
